# *Fusobacterium nucleatum* Facilitates Apoptosis, ROS Generation, and Inflammatory Cytokine Production by Activating AKT/MAPK and NF-*κ*B Signaling Pathways in Human Gingival Fibroblasts

**DOI:** 10.1155/2019/1681972

**Published:** 2019-10-13

**Authors:** Wenyan Kang, Zhilong Jia, Di Tang, Zhanwei Zhang, Hui Gao, Kunlun He, Qiang Feng

**Affiliations:** ^1^Shandong Provincial Key Laboratory of Oral Tissue Regeneration & Shandong Engineering Laboratory for Dental Materials and Oral Tissue Regeneration, School of Stomatology, Shandong University, Jinan, Shandong 250012, China; ^2^Department of Human Microbiome, School of Stomatology, Shandong University, Jinan, Shandong 250012, China; ^3^Department of Periodontology, School of Stomatology, Shandong University, Jinan, Shandong 250012, China; ^4^Beijing Key Laboratory for Precision Medicine of Chronic Heart Failure, Chinese PLA General Hospital, Beijing 100853, China; ^5^Department of Stomatology, Weifang People's Hospital, Weifang, Shandong 261042, China; ^6^State Key Laboratory of Microbial Technology, Shandong University, Qingdao, Shandong 266237, China

## Abstract

*Fusobacterium nucleatum* (*F. nucleatum*) plays key roles in the initiation and progression of periodontitis. However, the pathogenic effect of *F. nucleatum* on human oral tissues and cells has not been fully evaluated. In this study, we aimed to analyze the pathogenic effects of *F. nucleatum* on human gingival fibroblasts (GFs) and clarify the potential mechanisms. RNA-sequencing analysis confirmed that *F. nucleatum* significantly altered the gene expression of GF as the stimulation time increased. Cell counting and EdU-labeling assays indicated that *F. nucleatum* inhibited GF proliferation and promoted cell apoptosis in a time- and dose-dependent manner. In addition, cell apoptosis, intracellular reactive oxygen species (ROS) generation, and proinflammatory cytokine production were dramatically elevated after *F. nucleatum* stimulation. Furthermore, we found that the AKT/MAPK and NF-*κ*B signaling pathways were significantly activated by *F. nucleatum* infection and that a large number of genes related to cellular proliferation, apoptosis, ROS, and inflammatory cytokine production downstream of AKT/MAPK and NF-*κ*B signaling pathways were significantly altered in *F. nucleatum*-stimulated GFs. These findings suggest that *F. nucleatum* inhibits GF proliferation and promotes cell apoptosis, ROS generation, and inflammatory cytokine production partly by activating the AKT/MAPK and NF-*κ*B signaling pathways. Our study opens a new window for understanding the pathogenic effects of periodontal pathogens on the host oral system.

## 1. Introduction

Periodontal diseases are prevalent globally, with approximately 60% of the adult population suffering from mild, moderate, or aggressive periodontitis [1, 2]. As a chronic inflammatory disease, periodontitis is characterized by the destruction of the gingiva, periodontal ligament, cementum, and alveolar bone and is initiated by the invasion of specific oral pathogens that colonize dental plaque biofilms on the tooth surface [3–5]. The pathogenesis of periodontitis is complex, and excessive tissue destruction occurs as a result of interactions between pathogenic bacteria and the host immune inflammatory response [3]. Previous studies have confirmed that periodontitis is commonly caused by gram-negative bacterial infection such as *Porphyromonas gingivalis* (*P. gingivalis*), *Tannerella forsythia*, and *Aggregatibacter actinomycetemcomitans* [6–9]. However, *Fusobacterium nucleatum* (*F. nucleatum*), which is also a fundamental periodontal pathogen, has not been extensively studied in the field of periodontics, and the pathogenicity of *F. nucleatum* has not been extensively studied in oral tissues and cells.

*F. nucleatum*, as a bridging bacterium, transfers critical periodontal pathogens to periodontal infectious sites and recruits and activates local immune cells, which results in tooth-supporting tissue destruction [10, 11]. *F. nucleatum* has been identified as a high-frequency pathogen in periodontal disease [12] and many other infectious diseases, such as ventriculitis and brain abscesses [13], liver abscesses [14, 15], lung abscesses [16], septicemia-related infections [17], pelvic inflammatory disease [18], and intrauterine infections [19–21]. *F. nucleatum* attacks host tissues and obstructs the healing of damaged oral tissues by secreting large amounts of ammonia and butyrate [22, 23] and accelerates the initiation and progression of colorectal cancer by promoting tumor cell proliferation [24]. However, the effects of *F. nucleatum* on gingival fibroblast (GF) proliferation and apoptosis have not been reported.

The host response to pathogenic invasion is the determining factor of human health. As the first line of physical and chemical defense against infection, gingival epithelial cells release antimicrobial peptides such as human *β*-defensins to repress plaque biofilm formation, and activate innate and adaptive immune responses to protect the host from injury [25, 26]. Pathogens penetrate the connective tissue, directly damage the periodontal supporting tissues, and then induce the destruction of the integrity of the gingival epithelium [5, 27]. Human GFs, the predominant cell type of the periodontal connective tissue, are activated and recruited to sites of infection to regulate the occurrence and development of diseases [28–30]. It has been reported that GFs directly act on oral pathogens to defend against the progression of the infection by secreting various types of cytokines and chemokines, such as interleukin- (IL-) 1*β*, IL-6, IL-8, and tumor necrosis factor- (TNF-) *α* [31, 32]. Cytokine production is a crucial component for host defense against pathogenic invasion [31, 32]. A previous study confirmed that GFs showed no tolerance to bacterial stimulation and could continuously respond to exogenous stimuli and produce high levels of inflammatory cytokines [33]. However, the understanding of the pathogenic effects of *F. nucleatum* on inflammatory cytokine production by GFs and the potential mechanism has not been completely elucidated.

It has been reported that bacterial infection induces the mitochondrial electron transport for aerobic respiration and then dramatically elevates the intracellular reactive oxygen species (ROS) levels, which play critical roles in regulating cellular proliferation, apoptosis, and inflammatory response [34–36]. *F. nucleatum* has been shown to induce bladder cancer cell apoptosis through mediating ROS generation and mitochondrial dysfunction [34]. *F. nucleatum* enhances proinflammatory cytokine production by causing the impairment of autophagic flux in Caco-2 cells [35]. However, the effects of *F. nucleatum* on GF ROS generation remain unclear.

In this study, we aimed to explore the comprehensive gene expression profile of GFs over an *F. nucleatum* stimulation time course. We validated the effects of *F. nucleatum* on GF proliferation, apoptosis, intracellular ROS generation, and inflammatory cytokine production by biological experiments. Furthermore, we investigated the potential mechanism by which *F. nucleatum* regulated the biological properties of GFs according to the significantly enriched signaling pathways.

## 2. Materials and Methods

### 2.1. Human Subjects and Ethical Statements

This study was approved by the Medical Ethical Committee of the Stomatology School, Shandong University (Protocol Number: 20170101). Five healthy individuals aged 30-35 who underwent impacted tooth extraction at the Department of Oral and Maxillofacial Surgery, Stomatology Hospital of Shandong Province, were recruited. All individuals were informed about the research project and signed the informed consent form according to the Helsinki Declaration of 1975.

### 2.2. Cell Isolation and Culture

The excised gingival tissues were immediately immersed in Dulbecco's modified Eagle's medium (DMEM, HyClone, Logan, UT, USA) with 5% antibiotics (100 U/ml penicillin and 100 mg/ml streptomycin, Sigma-Aldrich, St Louis, MO, USA) and quickly transferred to the laboratory. Then, the free gingival tissue samples were washed, and the epithelium was removed. The samples were minced into small fragments of approximately 1-3 mm^2^ and digested for 2 h at 37°C by 3 mg/ml collagenase I (Sigma Aldrich) and 4 mg/ml Dispase II (Invitrogen, Carlsbad, CA, USA). The primary GFs were cultured with DMEM containing 20% fetal bovine serum (FBS, Biological Industries, Kibbutz, Israel) at 37°C in a humidified atmosphere of 5% CO_2_. Cells were fed fresh medium every three days until the cell monolayer reached 80-90% confluence. GFs were trypsinized and passaged at a dilution ratio of 1 : 3 to expand the culture in 10% FBS medium. The fourth passage cells were used for the following experiments, and cells were cultured in 10% FBS DMEM in all experiments.

### 2.3. RNA-Sequencing (RNA-Seq) Analysis

A total of 54 samples from 5 individuals were sequenced to analyze the gene expression at the whole genome level at BGI (Beijing Genomics Institute, Shenzhen, China) by RNA-seq (each individual sample included 1 sample at time point 0 h; 5 control samples at 2 h, 6 h, 12 h, 24 h, and 48 h with no *F. nucleatum* treatment; and 5 experimental samples with *F. nucleatum* stimulation at multiplicity of infection (MOI) of 100 at the same time points; sufficient RNA could not be extracted from one treated sample at the 12 h time point). Total RNA was isolated, evaluated for quality, reverse-transcribed to cDNA, and sequenced on the BGISEQ-500 platform. After a quality control (QC) step (Agilent 2100 Bioanalyzer, Santa Clara, California, USA), the clean reads were mapped to the reference genome (GRCh38) via hierarchical indexing for spliced alignment of transcripts (HISAT) (v2.0.4) [37]. Bowtie 2 (v2.2.5) [38] was used to map the clean reads to the reference transcripts, and the gene expression level for each sample was calculated by RSEM [39]. The correlation among all samples was detected by principal component analysis (PCA). Based on the gene expression level, DESeq2 and PoissonDis algorithms were used to detect the differentially expressed genes (DEGs) between the control groups and the *F. nucleatum*-treated groups by an absolute of log base 2 of fold change in DEGs ≥ 1 and an adjusted *P* value (%) < 5%. Gene ontology (GO) was used for screening and annotating DEGs. WEGO software [40] was used to generate the GO functional classification file. Pathway enrichment analysis of DEGs was performed based on the Kyoto Encyclopedia of Genes and Genomes (KEGG) database [41]. A Venn diagram was used to show the overlapping genes among the DEGs of each group. The protein-protein interaction network was generated by the STRING [42] website with default parameters. The Pathview [43] website was used to analyze the signaling pathway activation after *F. nucleatum* stimulation. In addition, Venn diagrams and heat maps were used to present the expression signatures of the DEGs involved in the GO biological processes, including cell proliferation (GO: 0008283), apoptotic process (GO: 0006915), response to reactive oxygen species (GO: 0000302), and defense response (GO: 0006952). The RNA sequence data have been deposited in the NCBI Gene Expression Omnibus (GEO, http://www.ncbi.nlm.nih.gov/geo/) and are accessible through GEO series accession number GSE118691.

### 2.4. Cell Proliferation Assay

GFs were seeded in 6-well (1 × 10^5^ cells/well) plates in growth medium with 10% FBS. Cells were left untreated or incubated with *F. nucleatum* at multiplicities of infection (MOIs) (*F. nucleatum* : cell of 10 : 1, 50 : 1, 100 : 1, 200 : 1, and 400 : 1). Cells were counted every other day using a hemocytometer (Corning, Corning, NY, USA) or an automated cell counter (Countstar, Shanghai, China). To assess the cell proliferation rate, cells were inoculated in 24-well plates (5 × 10^4^ cells/well) and treated with the indicated concentration of *F. nucleatum*. The 5-ethynyl-2′-deoxyuridine labeling assay was used to evaluate the cell proliferation rate according to the instructions of an EdU Apollo DNA in vitro kit (RiboBio, Guangzhou, China). The experiment was performed in sextuplicate and repeated three times.

### 2.5. Cell Apoptosis and Viability Analysis

Cell apoptosis was analyzed according to the instructions of an Annexin V-FITC/PI kit (Dojindo, Kumamoto, Japan). GFs were seeded in 6-well plates (2 × 10^5^cells/well) and stimulated with different densities of *F. nucleatum* for 2, 6, 12, 24, 36, and 48 h. Afterward, cells were trypsinized, washed with phosphate-buffered saline (PBS), stained with a PI-conjugated anti-annexin V antibody for 15 min at room temperature in the dark, and then subjected to flow cytometry; the data were analyzed by Accuri C6 Plus software (Becton Dickinson, Franklin Lakes, NJ, USA). Cell viability was detected by trypan blue (Solarbio) staining. GFs treated with 4% formaldehyde were used as a positive control, and untreated cells were used as a negative control. Trypan blue was added to the monolayers at a 1 : 10 dilution and incubated for 10 min. The cells were observed immediately under the microscope and photographed (Olympus, Tokyo, Japan). The experiment was performed in triplicate and repeated three times.

### 2.6. Measurement of Intracellular ROS

Intracellular ROS levels were detected by a 2′,7′-dichlorofluorescein diacetate (DCFH-DA) assay. GFs were seeded in 6-well plates and treated with *F. nucleatum* at the above-mentioned MOIs for 2, 6, 12, 24, 36, and 48 h. Cells were incubated with DCFH-DA (1 : 1000) for 20 min at 37°C in a cell incubator in the dark. Flow cytometry (Becton Dickinson) was used to determine the intracellular ROS production. The flow cytometry data were analyzed by Accuri C6 Plus software. Each experiment was performed in triplicate and repeated three times.

### 2.7. RNA Isolation and Quantitative Real-Time Polymerase Chain Reaction (qRT-PCR)

GFs were seeded in 6-well plates and cultured with *F. nucleatum*. GFs were collected at 6 different time points (0 h, 2 h, 6 h, 12 h, 24 h, and 48 h), and total RNA was extracted with TRIzol® (CWBIO, Beijing, China). The mRNA concentration was determined using an ultramicro spectrophotometer (Thermo Fisher Scientific, Waltham, MA, USA). One microgram of mRNA was reverse-transcribed to cDNA using a HiFiScript cDNA Synthesis kit (CWBIO). qRT-PCR was performed with an UltraSYBR Mixture (CWBIO) on a LightCycler 96 Real-Time PCR System (Roche, Basel, Switzerland) in triplicate. Briefly, the hot start enzyme was activated (95°C for 10 min), and the cDNA was then amplified for 45 cycles of denaturation at 95°C for 10 s, annealing at 60°C for 30 s and extension at 72°C for 32 s. Data were analyzed using the 2^(-*ΔΔ*Ct)^ method. The sequences of the primers for amplification are shown in Supplementary [Supplementary-material supplementary-material-1]. The experiment was performed in triplicate and repeated three times.

### 2.8. Enzyme-Linked Immunosorbent Assay (ELISA)

GFs were seeded in 6-well plates and treated with *F. nucleatum* at an MOI of 100 : 1. Cell culture supernatants were collected and centrifuged at 12,000 rpm for 5 min at 4°C. The levels of secreted IL-6, IL-8, IL-1*β*, and TNF-*α* protein were measured by ELISA (BioLegend, San Diego, CA, USA) according to the manufacturer's instructions. The optical density values were measured by a microplate reader at 450 nm and 570 nm, and the 570 nm values were subtracted from the absorbance at 450 nm in the subsequent data analysis. The experiment was performed in triplicate and repeated three times.

### 2.9. Western Blot Assay

GFs with or without *F. nucleatum* stimulation were harvested with a RIPA lysis buffer containing 1% protease inhibitors and 1% phosphatase inhibitors (Solarbio, Beijing, China). Protein concentrations were measured according to bicinchoninic acid (BCA) assays, and proteins (20 *μ*g/lane) were separated to 10% sodium dodecyl sulfate-polyacrylamide gel electrophoresis (SDS-PAGE) gels and transferred to polyvinylidene fluoride (PVDF) membranes (Millipore, Billerica, MA, USA). Membranes were blocked with 5% nonfat milk for 1 h, incubated with primary antibodies overnight at 4°C, and then incubated with horseradish peroxidase-conjugated secondary antibodies (1 : 10 000; Proteintech, Chicago, IN, USA) for 1 h at room temperature. The protein bands were visualized with enhanced chemiluminescence reagents (Millipore) and scanned using an extra-sensitive imager (Amersham Imager 600; GE Healthcare Life Sciences, Pittsburgh, PA, USA). ImageJ 1.44 software (NIH, Bethesda, Maryland, USA) was used to quantify the protein expression levels. The primary antibodies and dilution ratio were as follows: rabbit anti-NF-*κ*B p65 (1 : 10000; Abcam, Cambridge, UK), rabbit anti-phospho-NF-*κ*B p65 (1 : 1000; Abcam), rabbit anti-I*κ*B*α* (1 : 5000; Abcam), rabbit anti-phospho-I*κ*B*α* (1 : 1000;Abcam), rabbit anti-p38 (1 : 1000; Cell Signaling Technology, Danvers, MA, USA), rabbit anti-phospho-p38 (1 : 1000; Cell Signaling Technology), rabbit anti-JNK (1 : 1000; Cell Signaling Technology), rabbit anti-phospho-JNK (1 : 1000; Cell Signaling Technology), rabbit anti-ERK1/2 (1 : 1000; Cell Signaling Technology), rabbit anti-phospho-ERK1/2 (1 : 1000; Cell Signaling Technology), rabbit anti-AKT (1 : 1000; Cell Signaling Technology), rabbit anti-phospho-AKT (1 : 1000; Cell Signaling Technology), rabbit anti-p53 (1 : 1000; Abcam), and rabbit anti-phospho-p53 (1 : 1000;Abcam). The experiment was performed in triplicate and repeated three times.

### 2.10. Cell Immunocytochemistry Assay

To detect *F. nucleatum*-induced nuclear translocation of NF-*κ*B p65 and p-p65, GFs were seeded in coverslip containing 24-well plates at a density of 2 × l0^4^ cells/well and treated with or without *F. nucleatum* for 5 min to 120 min. Cells were fixed with 4% paraformaldehyde and blocked with 10% normal goat serum in PBS for 1 h. Then, cells were incubated with primary antibodies against p65 and p-p65 overnight at 4°C. The next day, the cells were incubated with an Alexa Fluor 594-conjugated goat anti-rabbit IgG secondary antibody (1 : 500) in the dark for 1 h, and the nuclei were visualized using 2-(4-amidinophenyl)-6-indolecarbamidine dihydrochloride (DAPI) for 5 min. The images were captured by fluorescence microscopy (Olympus BX53, Tokyo, Japan). The experiment was performed in triplicate and repeated three times.

### 2.11. Statistical Analysis

All data were expressed as the mean ± standard deviation (SD). Tests were analyzed using GraphPad Prism software (version 6, Software MacKiev, Boston, MA, USA), and differences among more than two groups were analyzed by one-way or two-way ANOVA followed by Tukey's honestly significant difference (HSD) comparison test. Variance between two groups was compared by a multiple *t*-test. *P* < 0.05 was considered indicative of statistical significance.

## 3. Results

### 3.1. RNA-Seq Analysis of *F. nucleatum*-Stimulated GFs over Time

To better understand the global responses of GFs to *F. nucleatum* infection over time, we performed a genome-wide transcriptome analysis by RNA-seq to determine the global changes in gene expression. A total of 100 Gb of sequence data was generated from 54 samples, and 23.90 million reads for each sample were generated on average (Supplementary [Supplementary-material supplementary-material-1]). After QC, the clean reads were mapped to the reference genomes and transcripts with mapping percentages of 94.40% and 87.67%, respectively (Supplementary [Supplementary-material supplementary-material-1] and Supplementary [Supplementary-material supplementary-material-1]). A total of 18691 genes were detected, and the expression levels were calculated with RSEM (data can be obtained through GSE118691). The correlation analysis of the PCA on the whole-genome gene expression levels showed that the normal cells were relatively stable, while the *F. nucleatum-*stimulated GFs gradually deviated from normal conditions as the stimulus time increased (Supplementary [Supplementary-material supplementary-material-1]). These results suggest that the effect of *F. nucleatum* stimulation on GFs may accumulate over time.

**Figure 1:**
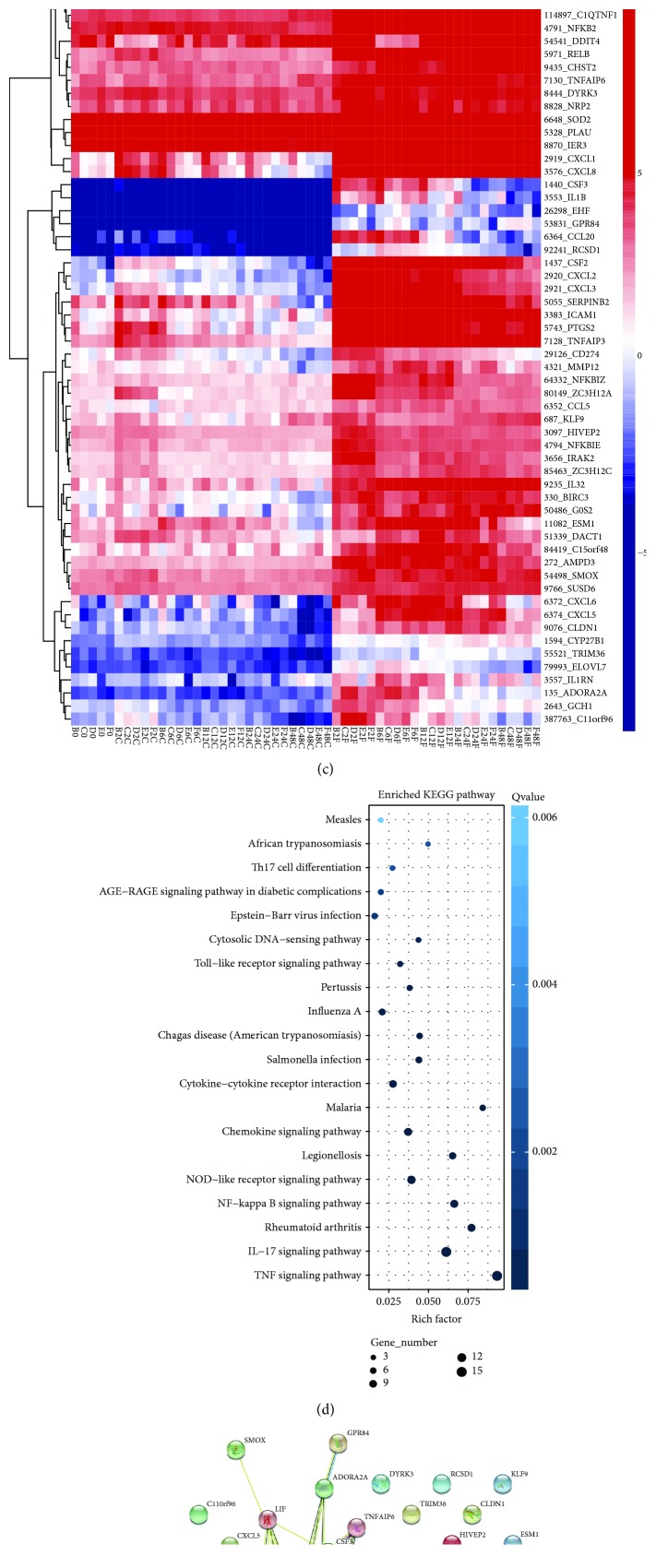
RNA-sequencing analysis of GFs stimulated with *F. nucleatum* (MOI of 100). (a) The number of DEGs in 54 samples after *F. nucleatum* stimulation at 2 h, 6 h, 12 h, 24 h, and 48 h. (b) Venn diagram summarizing the overlapping DEGs among the five time points. (c) Heat map of the 62 overlapping DEGs. (d) KEGG enrichment analysis of the 62 overlapping DEGs. (e) Network of 62 overlapping DEGs after *F. nucleatum* stimulation at 2 h, 6 h, 12 h, 24 h, and 48 h.

To characterize the DEGs influenced by *F. nucleatum*, the gene expression profiles of normal GFs versus infected cells at 2, 6, 12, 24, and 48 h were compared at each time point. Approximately 228, 374, 616, 1208, and 1334 genes were found to be upregulated, and 28, 147, 448, 1302, and 2145 genes were downregulated at 2, 6, 12, 24, and 48 h, respectively (Figure 1(a)). The overlap analysis of the DEGs among the 5 time points identified that 62 genes were differentially expressed between nonstimulated and *F. nucleatum*-stimulated GFs across the entire time course, and the number of DEGs continuously increased as the stimulation time increased (Figure 1(b)). These results suggest that the response of GFs to *F. nucleatum* infection increases over time, and a longer duration *F. nucleatum* stimulation might induce more side-effects than a short duration infection.

The heat map showed that the gene expression levels of 62 DEGs were upregulated and that some of these genes, such as IL-6, IL-1*β*, CCL2, CXCL2, CXCL8, and CSF3, are closely associated with host defense responses to bacterial infection (Figure 1(c)). The KEGG annotation showed that the top 5 enriched pathways of these DEGs are immune-associated, namely, the TNF-signaling pathway, IL-17 signaling pathway, rheumatoid arthritis, NF-*κ*B signaling pathway, and NOD-like receptor signaling pathway (Figure 1(d)). The protein-protein interaction network of 62 DEGs showed the potentially complex interaction relationship among these genes (Figure 1(e)).

The GO analysis confirmed that the 62 DEGs are involved in host defense to bacterial infection and cell growth pathways, such as cellular process, response to stimulus, regulation of biological process, immune system process, response to bacterium, and cell proliferation (Supplementary [Supplementary-material supplementary-material-1]), and the top five enriched biological process GO terms for the 62 DEGs are shown in Supplementary [Supplementary-material supplementary-material-1].

### 3.2. *F. nucleatum* Inhibits the Proliferation of GFs

**Figure 2:**
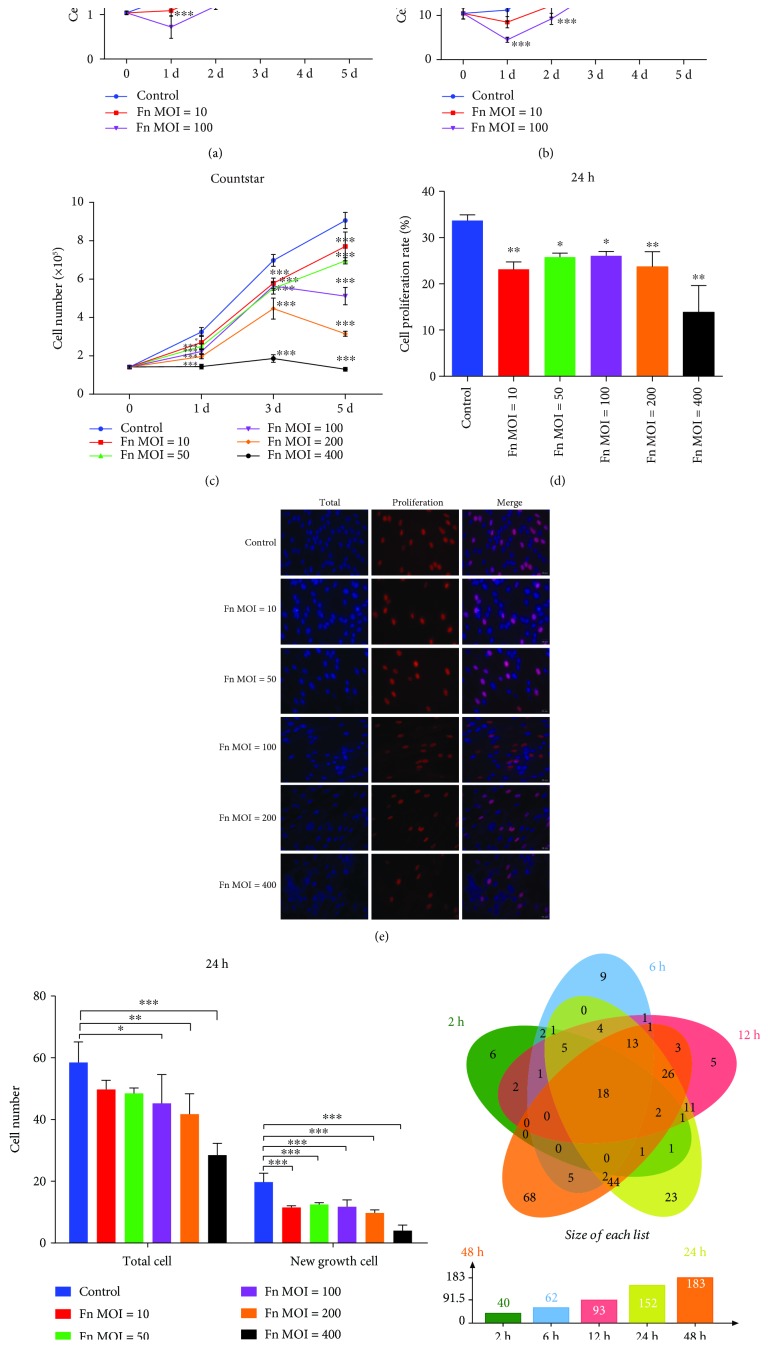
Effects of *F. nucleatum* on cell proliferation. Cell numbers of GFs detected by an electronic cell counter (a) and hemocytometer (b) with or without *F. nucleatum* stimulation at MOIs of 10 and 100 (*n* = 6). (c) Number of GFs detected by electronic cell counter after *F. nucleatum* stimulation (MOIs of 0, 10, 50, 100, 200, and 400, *n* = 6). (d) Cell proliferation rate of GFs detected by EdU assay (*n* = 6). (e) EdU assay of GFs after *F. nucleatum* stimulation (MOIs of 0, 10, 50, 100, 200, and 400) at 24 h. Scale bar: 20 *μ*m. NC: negative control; PC: positive control. (f) Statistical results of total and new cell growth after EdU labeling (*n* = 6). (g) Venn diagram of cell proliferation-related DEGs from the GO biological process analysis among the five time points. All data are shown as the mean ± SD. Statistical analyses were performed by one-way (d) and two-way (a, b, c, f) ANOVA with Tukey's multiple-comparison test. ^∗^*P* < 0.05, ^∗∗^*P* < 0.01, and ^∗∗∗^*P* < 0.001 compared with the Control.

To study the effect of *F. nucleatum* on the proliferation of GFs, we applied a cell counting assay and EdU-labeling assay to quantify the cell proliferation rate over 5 days of *F. nucleatum* stimulation at MOIs of 10 and 100. The cell counting assay showed that *F. nucleatum* significantly inhibited the proliferation of GFs in a time- and dose-dependent manner (Figures 2(a) and 2(b)). To further test the inhibitory effect of different *F. nucleatum* concentrations, GFs stimulated with *F. nucleatum* at MOIs of 10, 50, 100, 200, and 400 were quantified at 3 time points over 5 days. The results showed that the inhibitory effect aggrandized with the increase of *F. nucleatum* concentration, and the high concentration of *F. nucleatum* (MOI of 400) directly blocked the cell growth (Figure 2(c)). The EdU-labeling assay demonstrated that *F. nucleatum* significantly decreased the number of GFs and reduced the cell proliferation rate after 24 h of stimulation (Figure 2(d)–2(f)). The DEGs of the 54 samples enriched in the cell proliferation pathway (GO: 0008283) were compared using an overlap analysis, and the results indicated that the number of cell proliferation-related DEGs continuously increased as the *F. nucleatum* stimulation time increased; 40, 62, 93, 152, and 183 genes were detected at 2 h, 6 h, 12 h, 24 h, and 48 h, respectively, and 18 DEGs, including CCL2, SOD2, and NFKBIA, were consistently upregulated across all the time points (Figure 2(g) and Supplementary [Supplementary-material supplementary-material-1]). The above results suggest that *F. nucleatum* dose- and time-dependently regulates cell proliferation-related genes and inhibits the proliferation of GFs.

### 3.3. *F. nucleatum* Promotes the Apoptosis of GFs

**Figure 3:**
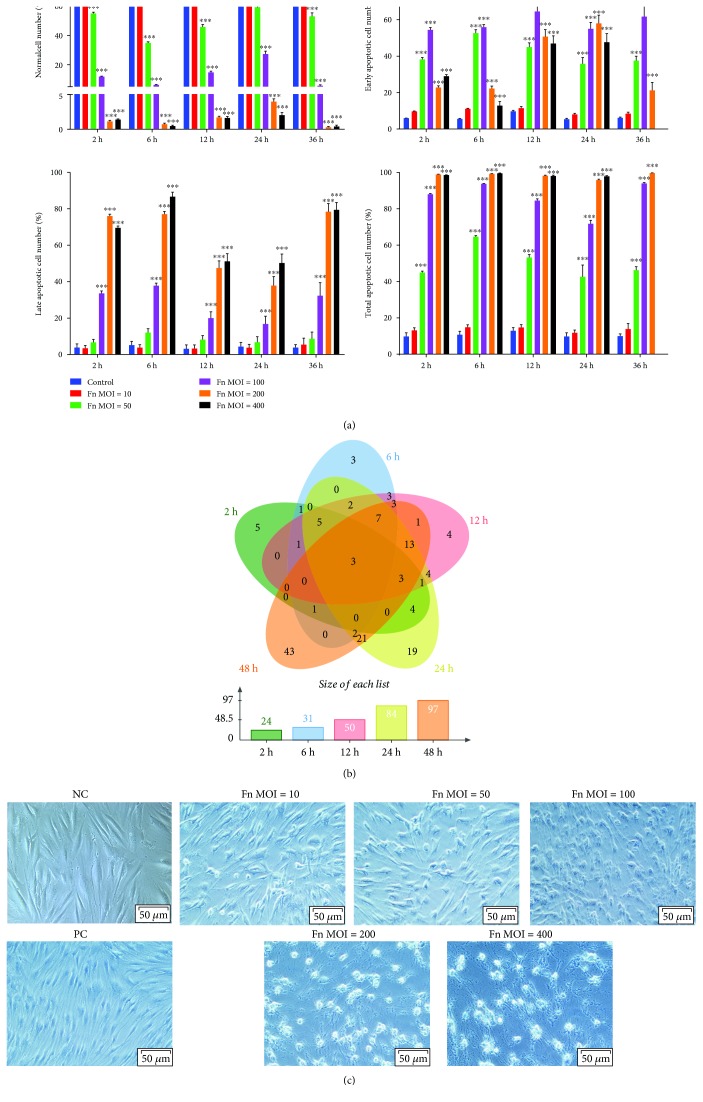
Effect of *F. nucleatum* on GF apoptosis. (a) Statistical analysis of GF apoptosis with *F. nucleatum* stimulation (MOIs of 0, 10, 50, 100, 200, and 400) at 2 h, 6 h, 12 h, 24 h, and 36 h. The histogram represents the mean ± SD (*n* = 3). (b) Venn diagram of apoptosis-related DEGs from the GO biological process analysis of the five paired comparisons. (c) Trypan blue staining results of GFs without or with *F. nucleatum* stimulation at MOIs of 10, 50, 100, 200, and 400 at 24 h. Scale bar: 50 *μ*m. NC: negative control; PC: positive control. Statistical analyses were performed by two-way ANOVA with Turkey's multiple-comparison test. ^∗∗∗^*P* < 0.001 compared with the control at each time point.

To further study the reason for the proliferation inhibition of GFs, the apoptosis rates of *F. nucleatum*-stimulated GFs were detected by flow cytometry. The results indicated that the low concentration of *F. nucleatum* (MOIs of 10) showed no influence on GF apoptosis, while high concentration of *F. nucleatum* (MOIs of 50, 100, 200, and 400) significantly decreased the number of normal cells and increased the number of apoptosis cells at 2 h stimulation and maintained the similar apoptosis trend to 36 h. At MOIs of 50 and 100, the number of early apoptotic cells was greater than the number of late apoptotic cells at the 2, 6, 12, 24, and 36 h *F. nucleatum* stimulation, while at the MOIs of 200 and 400, the number of late apoptotic GFs was larger than the early apoptotic cells (Figure 3(a) and Supplementary [Supplementary-material supplementary-material-1]). The DEGs of the 54 samples enriched in the apoptotic process (GO: 0006915) were compared using an overlap analysis, and the results suggested that 24, 31, 50, 84, and 97 apoptosis-related genes were differentially expressed at 2 h, 6 h, 12 h, 24 h, and 48 h, respectively (Figure 3(b)). Three apoptosis-related genes, CSF2, PTGS2, and SOD2, were continuously upregulated across the 5 time points (Figure 3(b) and Supplementary [Supplementary-material supplementary-material-1]). A trypan blue staining assay indicated that *F. nucleatum* dose-dependently increased the number of nonviable cells, which were stained blue, and the cell morphology changed from long spindle to round, and fewer live cells were detected after *F. nucleatum* stimulation at MOIs of 200 and 400 (Figure 3(c)). These results suggest that *F. nucleatum* might inhibit the proliferation of GFs by dose-dependently promoting GF apoptosis and time-dependently upregulating a group of apoptosis process-related genes.

### 3.4. *F. nucleatum* Promotes Intracellular ROS Generation in GFs

**Figure 4:**
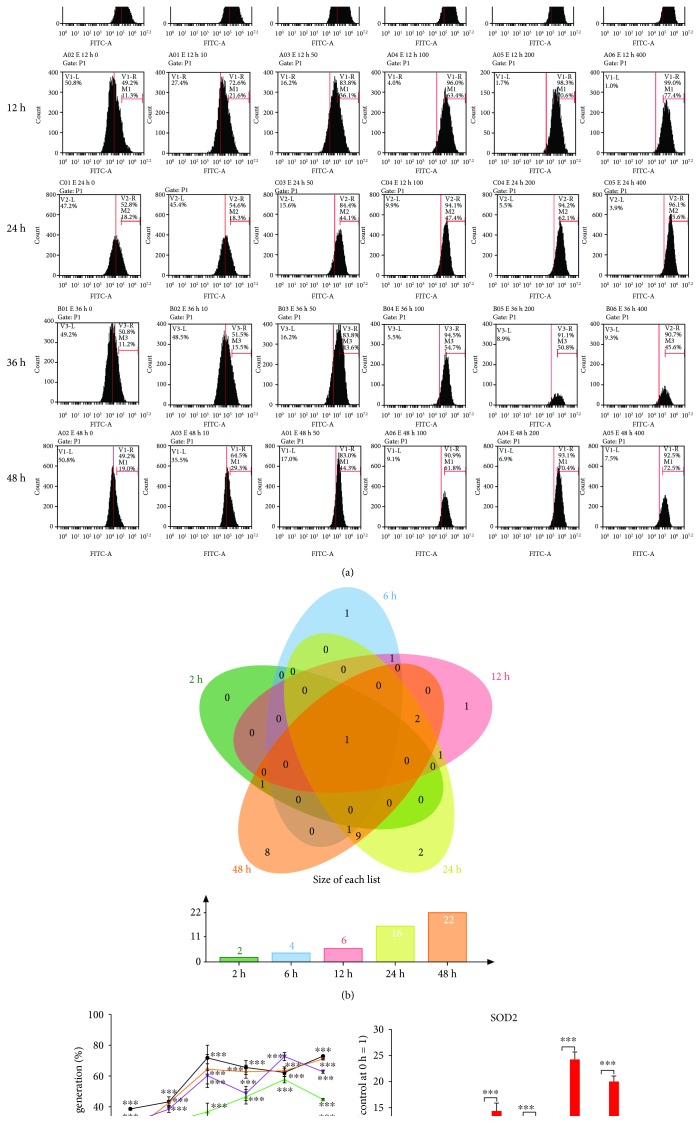
Effects of *F. nucleatum* on ROS generation in GFs. (a) Flow cytometry analysis of ROS production with *F. nucleatum* stimulation (MOIs of 0, 10, 50, 100, 200, and 400) at 2 h, 6 h, 12 h, 24 h, and 36 h. (b) Venn diagram of ROS production-related DEGs from the GO biological process analysis after the five paired comparisons. (c) Statistical results of ROS generation by flow cytometry (*n* = 3). (d) The relative gene expression level of SOD2 by qRT-PCR. The histogram represents the mean ± SD. Statistical analyses were performed by two-way ANOVA with Turkey's multiple-comparison test (c) and multiple *t*-test (d). ^∗^*P* < 0.05 and ^∗∗∗^*P* < 0.001 compared with the control at each time point.

To investigate whether the alteration in GF biological properties is associated with intracellular ROS production, GFs were treated with *F. nucleatum* (MOIs of 10, 50, 100, 200, and 400) for 2, 6, 12, 24, 36, and 48 h, and the level of ROS was detected at the different stimulation times and doses. The flow cytometry analysis results showed that the level of intracellular ROS was significantly elevated in a dose- and time-dependent manner (Figures 4(a) and 4(c)). The DEGs of the 54 samples enriched in the response to reactive oxygen species (GO: 0000302) were compared using an overlap analysis, and the results indicated that the number of differentially expressed ROS production-related genes also increased in a time-dependent manner, and 2, 4, 6, 16, and 22 DEGs were detected after *F. nucleatum* stimulation (MOI of 100) at 2, 6, 12, 24, 36, and 48 h (Figure 4(b)). SOD2 is a unique DEG that was continuously upregulated across the 5 time points. The relative gene expression level of SOD2 is shown in Figure 4(d). In addition, SOD2 also acts as the main contributor to the regulation of the proliferation and apoptosis of GFs (Supplementary [Supplementary-material supplementary-material-1] and Supplementary [Supplementary-material supplementary-material-1]).

### 3.5. *F. nucleatum* Promotes GF Inflammatory Cytokine Production

**Figure 5:**
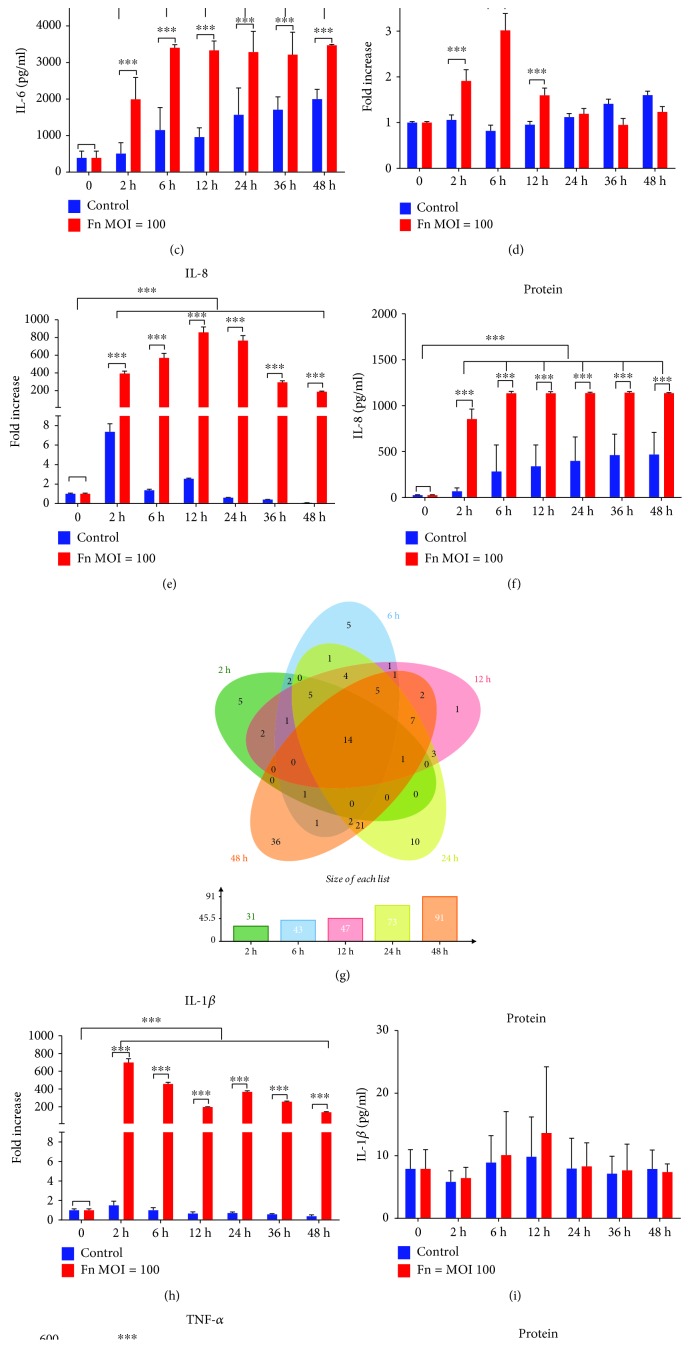
Effects of *F. nucleatum* on inflammatory cytokine production in GFs. TLR4 (a), TLR2 (d), IL-6 (b), IL-8 (e), IL-1*β* (h), and TNF-*α* (g) gene expression with *F. nucleatum* stimulation (MOIs of 0 and 100) from 0 to 48 h (*n* = 3). IL-6 (c), IL-8 (f), IL-1*β* (i), and TNF-*α* (k) protein expression with *F. nucleatum* stimulation (MOIs of 0 and 100) from 0 to 48 h (*n* = 3). (g) Venn diagram of the defense response-related DEGs among the five time points. The histogram represents the mean ± SD. Statistical analyses were performed by multiple *t*-test. ^∗^*P* < 0.05, ^∗∗^*P* < 0.01, and ^∗∗∗^*P* < 0.001.

The expression of Toll-like receptor- (TLR-) 2 and TLR4, which are closely related to inflammatory cytokine production, was analyzed by qRT-PCR. The expression of the TLR4 gene was elevated after *F. nucleatum* stimulation at 2 h, 12 h, 24 h, and 36 h, while showing no significant differences compared with the TLR4 expression in normal cells at 48 h (Figure 5(a)). The TLR2 gene expression level was increased in *F. nucleatum*-stimulated GFs at 2 h, 6 h, and 12 h, while showing no significant differences at 24 h and 48 h compared with that in nonstimulated GFs (Figure 5(d)). Compared with nonstimulated GFs at each time point, *F. nucleatum*-stimulated GFs showed significantly elevated gene expression levels of IL-6, IL-8, IL-1*β*, and TNF-*α* across the whole time course (Figures 5(b), 5(e), 5(h), and 5(j)); however, at the protein level, IL-6 and IL-8 were significantly increased after *F. nucleatum* stimulation, while IL-1*β* and TNF-*α* were not (Figures 5(c), 5(f), 5(i), and 5(k)). The DEGs of the 54 samples enriched in the defense response (GO: 0006952) were compared using an overlap analysis, which indicated that 31, 43, 47, 73, and 91 DEGs related to defense response were time-dependently increased after *F. nucleatum* stimulation; 14 overlapping DEGs were significantly upregulated among the 5 time points in the *F. nucleatum* stimulation groups (Figure 5(g) and Supplementary [Supplementary-material supplementary-material-1]).

### 3.6. *F. nucleatum* Activates GF Nuclear Factor-*κ*B (NF-*κ*B), Mitogen-Activated Protein Kinase (MAPK), and Protein Kinase B (AKT) Signaling Pathway

**Figure 6:**
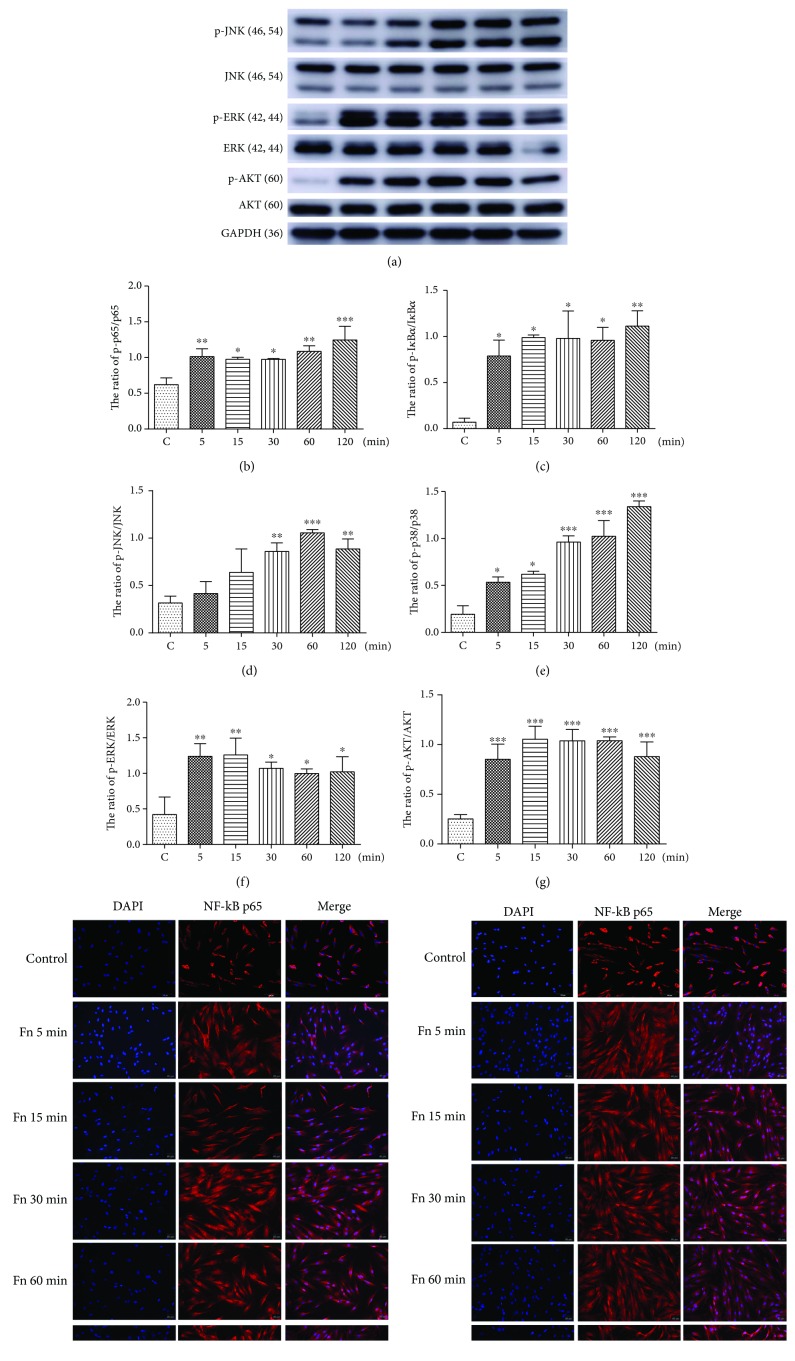
Effects of *F. nucleatum* on the activation of the NF-*κ*B, MAPK, and PI3K-AKT pathways in GFs. (a) The protein levels of p38, phosphorylated p38, JNK, phosphorylated JNK, ERK, phosphorylated ERK, NF-*κ*B p65, phosphorylated NF-*κ*B p65, I*κ*B*α*, phosphorylated I*κ*B*α*, AKT, and phosphorylated AKT were detected by western blotting. The relative level of phosphorylated NF-*κ*B p65/NF-*κ*Bp65 (b), phosphorylated I*κ*B*α*/I*κ*B*α* (c), phosphorylated JNK/JNK (d), phosphorylated p38/p38 (e), phosphorylated ERK/ERK (f), and phosphorylated AKT/AKT (g) was detected (*n* = 3). Immunofluorescence images of NF-*κ*B p65 (h) and phosphorylated NF-*κ*B p65 (i) in cells. The histograms represent means ± SD. Statistical analyses were performed using one-way ANOVA with Tukey's multiple-comparison test. ^∗^*P* < 0.05, ^∗∗^*P* < 0.01, and ^∗∗∗^*P* < 0.001 compared with the Control.

To further explore the mechanisms of *F. nucleatum*-induced biological process alteration in GFs, the cell proliferation, apoptosis, and immune response-related NF-*κ*B, MAPK, and AKT signaling pathways were analyzed by western blotting and immunofluorescence. The results showed that *F. nucleatum* increased the proportion of p-p65 to p65 and p-I*κ*B*α* to I*κ*B*α* after 5 min of stimulation and that this increase was maintained for 120 min (Figure 6(a)–6(c)). The immunofluorescence analysis of cells showed that p65 and p-p65 translocation from the cytoplasm to the nucleus also increased continuously after *F. nucleatum* stimulation from 5 min to 120 min (Figures 6(h) and 6(i)). These results indicate that *F. nucleatum* activates the NF-*κ*B signaling pathway by increasing the phosphorylation levels of p65 and I*κ*B*α* and increasing the nuclear-to-cytoplasmic ratio of NF-*κ*B p65 and NF-*κ*B p-p65 levels. Furthermore, we confirmed that *F. nucleatum* significantly activated AKT/MAPK signaling pathways by increasing the phosphorylation levels of AKT, ERK, JNK, and p38 (Figures 6(a) and 6(d)–6(g)). The protein expression ratio of p-AKT/AKT and p-ERK/ERK was elevated after *F. nucleatum* stimulation at 5 min and maintained at a high level until 120 min (Figures 6(f) and 6(g)). In addition, the relative value of p-JNK/JNK was increased after 30 min of *F. nucleatum* stimulation (Figure 6(d)), and the phosphorylation level of p38 was significantly increased after 5 min of stimulation with *F. nucleatum* (Figure 6(e)). Furthermore, the protein level of p53 and phosphorylated p53 was detected, and the results indicated that *F. nucleatum* could not increase the protein expression ratio of p-p53/p53 after stimulation for 5 min to 120 min (Supplementary [Supplementary-material supplementary-material-1]).

### 3.7. Whole-Transcriptome Analysis of NF-*κ*B, MAPK, and PI3K-AKT Signaling Pathways in *F. nucleatum*-Stimulated GFs

**Figure 7:**
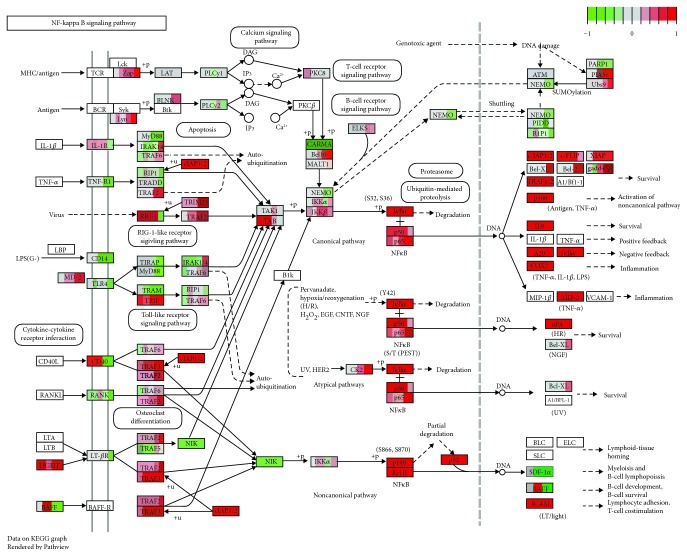
The Pathview analysis of the NF-*κ*B signaling pathway. Every box is divided into five parts on average, and each part represents the relative gene expression level of *F. nucleatum*-stimulated GFs at 2 h, 6 h, 12 h, 24 h, and 48 h from left to right. The red color indicates that the gene expression level is upregulated after *F. nucleatum* stimulation. The green color indicates that the gene level is downregulated after *F. nucleatum* stimulation. The white color indicates that the gene expression level is not influenced by *F. nucleatum* stimulation. The gene expression level is calculated by the log fold change in the *F. nucleatum* stimulation group relative to the control group at each time point.

To further clarify the correlation among cell proliferation, apoptosis, oxidative stress, cytokine production and AKT/MAPK, and NF-*κ*B signaling pathways, the DEGs induced by *F. nucleatum* stimulation from the 54 samples at each time point were integrated and visualized by Pathview. The results indicated that the DEGs were significantly enriched in the NF-*κ*B, PI3K-AKT, and MAPK signaling pathways, which suggest that these pathways are significantly activated by *F. nucleatum* stimulation at the gene expression level and are consistent with the results of our biological experiments (Figure 7, Supplementary [Supplementary-material supplementary-material-1] and Supplementary [Supplementary-material supplementary-material-1]). For the NF-*κ*B signaling pathway, we confirmed that most DEGs downstream of the NF-*κ*B signaling pathway were significantly upregulated by *F. nucleatum* stimulation, which is critical in regulating cell proliferation, apoptosis, ROS generation, and cytokine production (Figure 7). For example, cIAP1/2 and TRAF1/2 were elevated after *F. nucleatum* stimulation at 2 h, and Bcl-2 was upregulated at 12 h after *F. nucleatum* stimulation; these genes play key roles in cell proliferation and apoptosis. The gene expression levels of IL-8, cyclooxygenase 2 (COX2), A20, I*κ*B*α*, and MIP2 were significantly upregulated throughout the whole *F. nucleatum* stimulation (from 2 to 48 h); these genes are closely associated with GF intracellular ROS generation and inflammatory cytokine production (Figure 7 and Supplementary [Supplementary-material supplementary-material-1]). In addition, we found that a large number of *F. nucleatum*-stimulated DEGs were enriched in the PI3K-AKT signaling pathway (Supplementary [Supplementary-material supplementary-material-1]) and MAPK signaling pathways (Supplementary [Supplementary-material supplementary-material-1]), which are mainly involved in cell proliferation and apoptosis. Furthermore, we found that the PI3K-AKT and MAPK signaling pathways were upstream of the NF-*κ*B signaling pathway, which plays a synergetic role in regulating cell proliferation, apoptosis, ROS generation, and the inflammatory response.

## 4. Discussion

Characterized by gingival inflammation and periodontal supporting tissue destruction, periodontitis is prevalent among adults over 30 years old [44, 45]. Understanding the pathogenic mechanisms of oral pathogens in periodontitis and identifying risk factors are important for maintaining human periodontal health. Previous studies have provided in-depth insights into the pathogenic effects of *P. gingivalis* [46]. *F. nucleatum* often appears simultaneously with *P. gingivalis* at periodontal infection sites [47], while its pathogenic role in periodontal disease has not been comprehensively reported. This study is the first to illuminate the comprehensive effects of *F. nucleatum* on cell proliferation, apoptosis, ROS generation, and inflammatory cytokine production in GFs.

Our study confirms that *F. nucleatum* inhibits the proliferation and promotes the apoptosis of GFs, which is in contrast to the role *F. nucleatum* plays in colorectal cancer cell (CRC) proliferation. *F. nucleatum* has been reported to promote E-cadherin-expressing CRC proliferation by modulating the E-cadherin/*β*-catenin signaling pathway or activating TLR4 signaling to NF-*κ*B and upregulating the expression of microRNA-21 [24, 48]. Our transcriptomic analysis demonstrates that *F. nucleatum* alters the expression of proapoptotic genes in GFs by upregulating TRAILR2, NOXA, and PUMA expression and downregulating the substrates *α*-tubulin, actin, fodrin, and PARP (Supplementary [Supplementary-material supplementary-material-1]), which are crucial for the regulation of cell proliferation and apoptosis [49–55]. We corroborated the gene expression levels of TRAILR2, NOXA, PUMA, *α*-tubulin, actin, fodrin, and PARP by qRT-PCR, and the results indicated that *F. nucleatum* significantly upregulated TRAILR2 and NOXA at 2 to 48 h, while increased PUMA gene expression at 2, 24, and 48 h; *F. nucleatum* downregulated *α*-tubulin, actin, fodrin, and PARP gene expression at the long time stimulation at 12 h, 24 h, and 48 h, which were consistent with those of the RNA-seq analysis. However, at the 2 h and 6 h *F. nucleatum* stimulation, some disagreements between qRT-PCR and RNA-seq analysis were discovered in evaluating the gene level of actin and fodrin (Supplementary [Supplementary-material supplementary-material-1]). Moreover, *F. nucleatum* elevated the expression of Mcl-1, which ultimately induced apoptosis through bim-mediated mitochondrial apoptotic events (Supplementary [Supplementary-material supplementary-material-1]). *F. nucleatum* differentially regulates cell proliferation in GFs and CRCs, which indicates that the alteration of biological properties by *F. nucleatum* in normal human cells is quite different from that in tumor cell lines.

It has been reported that *F. nucleatum* stimulates ROS production and induces the tissue inflammatory response in TLR4-competent macrophages [56]. *F. nucleatum* induces IL-8, IL-1*β*, TNF-*α*, and ROS generation in Caco-2 colorectal adenocarcinoma cells by impairing autophagic flux [35]. In addition, recent studies have reported that in GFs, two NADPH oxidase isoforms, NOX1 and NOX2, are activated in response to *F. nucleatum* infection [57, 58]. However, the current study lacks the mechanism of ROS and inflammatory cytokine production in GFs after *F. nucleatum* stimulation. In this study, we confirm that *F. nucleatum* increases the production of ROS, activates TLR2 and TLR4 gene expression at early stimulation time points, and promotes the release of proinflammatory cytokines, such as IL-6 and IL-8 in GFs, which are concordant with the previous study. In addition, we found that *F. nucleatum* elevated the gene expression levels of IL-1*β* and TNF-*α*, but not the protein level. In gingival epithelial cells (GECs), *F. nucleatum* has been shown to activate the NLRP inflammasome and promote IL-1*β* secretion, which is different from our results in GFs [59, 60]. *F. nucleatum* differentially regulates IL-1*β* production in GFs and GECs, which indicates that the pathogenic mechanism of *F. nucleatum* in oral cells is quite complex, and it is necessary to clarify the mechanism of *F. nucleatum* in GFs.

Our genome-wide transcriptome analysis showed that *F. nucleatum* activates the NF-*κ*B, MAPK, and PI3K-AKT signaling pathways, which is consistent with our biological validation results through western blotting assays and immunofluorescence. In addition, we demonstrated that a large number of DEGs are enriched in the MAPK signaling pathway and that the JNK and p38 MAPK signaling pathways are upstream of the NF-*κ*B signaling pathway, which plays a key role in regulating cell proliferation, apoptosis, and inflammatory response [61–63]. The ERK MAPK and PI3K-AKT signaling pathways are significantly activated in GFs, and the expression levels of proliferation-related genes, such as Myc and Bcl-2, are dramatically varied after *F. nucleatum* stimulation, which are crucial in deciding the cell survival [64–67].

According to the Pathview analysis, we found that a larger number of DEGs that were induced by *F. nucleatum* stimulation downstream of NF-*κ*B and AKT/MAPK signaling pathways (Figure 7 and Supplementary Figures [Supplementary-material supplementary-material-1], [Supplementary-material supplementary-material-1]) are associated with cell proliferation, apoptosis, ROS generation, and production of inflammatory cytokines, such as bcl2, IL-8, COX2, JNK, and Mcl-1. We validated DEGs including COX2, IL-8, and bcl2 by qRT-PCR, and the results were consistent with those of the RNA-seq analysis. IL-8 is an important chemokine and plays key roles in the acute inflammatory response and various inflammatory diseases [68, 69]. COX2 is critical for the formation of prostaglandins and maintains ROS homeostasis under inflammatory conditions, which are pivotal in the pathogenesis of periodontitis [70, 71]. In the future, we will construct some gene knockout strains of *F. nucleatum* to further explore the mechanism of *F. nucleatum* in GFs.

## 5. Conclusions

**Figure 8:**
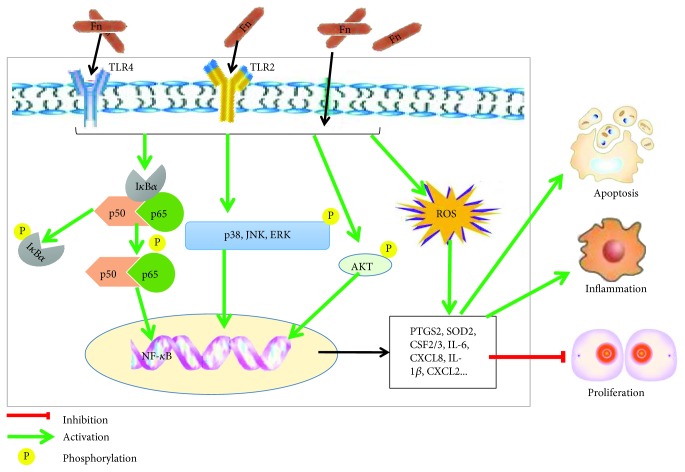
Putative mechanism for the effects of *F. nucleatum* on the biological processes in GFs. Schematic diagram depicting how *F. nucleatum* activates NF-*κ*B, MAPK, and AKT signaling pathways and induces cell proliferation, apoptosis, and inflammatory response. TLR4: Toll-like receptor 4; TLR2: Toll-like receptor 2; I*κ*B*α*: inhibitors of NF-*κ*B; AKT/PKB: protein kinase B; NF-*κ*B: nuclear factor kappa B; MAPK: mitogen-activated protein kinase; JNK: c-Jun N-terminal kinase; ERK: extracellular signal-regulated kinase.

Our study indicates that *F. nucleatum* alters the gene expression profiles of GFs in a time-dependent manner and plays multidimensional roles in regulating GF biological properties. *F. nucleatum* inhibits cell proliferation and facilitates cell apoptosis, ROS generation, and inflammatory cytokine production partly through the activation of AKT/MAPK and the NF-*κ*B signaling pathways in GFs. Our study opens a new window for understanding the pathogenic effects of periodontal pathogens on the host oral system. A schematic diagram of the effects of *F. nucleatum* on GFs and the potential pathogenic mechanism is shown in Figure 8.

## Data Availability

All raw RNA-seq data are accessible through GEO series accession number, GSE118691. Other data from Materials and Methods used to support the findings of this study are included in the article. If any other data are needed, please contact the corresponding author.

## Abbreviations

*F. nucleatum*: *Fusobacterium nucleatum*
GFs: Gingival fibroblasts
ROS: Reactive oxygen species
IL: Interleukin
TNF: Tumor necrosis factor
DMEM: Dulbecco's modified Eagle's medium
FBS: Fetal bovine serum
RNA-seq: RNA sequencing
QC: Quality control
DEGs: Differentially expressed genes
GO: Gene ontology
KEGG: Kyoto Encyclopedia of Genes and Genomes
PCA: Principal component analysis
PBS: Phosphate-buffered saline
MOI: Multiplicity of infection
EdU: 5-Ethynyl-2′-deoxyuridine
DCFH-DA: 2′,7′-dichlorofluorescein diacetate
qRT-PCR: Quantitative real-time polymerase chain reaction
ELISA: Enzyme-linked immunosorbent assay
BCA: Bicinchoninic acid
SDS-PAGE: Sulfate-polyacrylamide gel electrophoresis
PVDF: Polyvinylidene fluoride
DAPI: 2-(4-Amidinophenyl)-6-indolecarbamidine dihydrochloride
SD: Standard deviation
ANOVA: Analysis of variance
TLRs: Toll-like receptors
NF-*κ*B: Nuclear factor-*κ*B
MAPK: Mitogen-activated protein kinase
AKT: Protein kinase B
CRCs: Colorectal cancer cells
GECs: Gingival epithelial cells
HSD: Honestly significant difference.

## Supplementary Materials

Supplementary 1 Table 1: primer sequences for quantitative real-time PCR (qRT-PCR).

Supplementary 2 Table 2: the clean reads of 54 samples after RNA-seq.

Supplementary 3 Table 3: genome mapping ratio summary of 54 samples.

Supplementary 4 Table 4: gene mapping ratio summary of 54 samples.

Supplementary 5 Table 5: top five enriched GO terms in the biological process for the 62 DEGs.

Supplementary 6 Figure 1: PCA of 54 samples of RNA-seq analyses.

Supplementary 7 Figure 2: GO analysis of the 62 overlapped DEGs after *F. nucleatum* stimulation at 2 h, 6 h, 12 h, 24 h, and 48 h.

Supplementary 8 Figure 3: heat map summarizing the 18 overlapped DEGs from the GO biological process analysis after the five paired comparisons of cell proliferation.

Supplementary 9 Figure 4: (a) Flow cytometry analysis of cell apoptosis. (b) Heat map of the 3 overlapped DEGs from the GO biological process analysis after the five paired comparisons of cell apoptosis.

Supplementary 10 Figure 5: heat map of the 14 overlapping DEGs from the GO biological process analysis after the five paired comparisons of cell defense response.

Supplementary 11 Figure 6: (a) The protein levels of p53, phosphorylated p53 (p-p53). (b) The ratio of p-p53 and p53.

Supplementary 12 Figure 7: the Pathview analysis of the PI3K-AKT signaling pathway.

Supplementary 13 Figure 8: the Pathview analysis of MAPK signaling pathway.

Supplementary 14 Figure 9: the relative gene expression level of Bcl-2 (a), COX2(b), A20 (c), I*κ*B*α* (d), and MIP2 (e) by qRT-PCR.

Supplementary 15 Figure 10: the Pathview analysis of the apoptosis signaling pathway.

Supplementary 16 Figure 11: the relative gene expression level of TRAILR2 (a), NOXA (b), PUMA (c), tubulin (d), actin (e), fodrin (f), and PARP (g) by RT-PCR.
