# Supplementary material for: Fusobacterium nucleatum Facilitates Apoptosis, ROS Generation, and Inflammatory Cytokine Production by Activating AKT/MAPK and NF-κB Signaling Pathways in Human Gingival Fibroblasts
Source: Oxid Med Cell Longev. 2019 Oct 13;2019:1681972. doi: 10.1155/2019/1681972 (PMC6815639; doi:10.1155/2019/1681972)
Supplement: Supplementary 1 — Table 1: primer sequences for quantitative real-time PCR (qRT-PCR). [file 1681972.f1.docx]

**Supplementary Table 1:** Primers sequences for quantitative real-time PCR (qRT-PCR)

| Gene | Primer sequences | |
| --- | --- | --- |
|  | 5ˊ~3ˊForward | 5ˊ~3ˊReverse |
| GAPDH  TLR4  TLR2  IL-6  IL-8  IL-1β  TNF-α  Bcl2  Fodrin  α-Tublin  TRAILR2  NOXA  PUMA  PARP  A20  MIP2  Actin  COX2  IkBα | GCACCGTCAAGGCTGAGAAC  TGGTGTCCCAGCACTTCATC  TTATCCAGCACACGAATACACAG  ATAACCACCCCTGACCCAAC  TCAGAGACAGCAGAGCACAC  CTTTGAAGCTGATGGCCCTAAA  CCCAGGGACCTCTCTCTAATCA  GAACTGGGGGAGGATTGTGG  TCCTTGCTGACTTCCGTGAC  TGTTCACTGGTACGTTGGGG  TCGTGAGTATCTTGCAGCCC  CCCTTGGAAACGGAAGATGG  CTCTCGGTGCTCCTTCACTC  CCCCACGACTTTGGGATGAA  CTCGGGGAGAAGCCTATGAG  AGATCAATGTGACGGCAGGG  CTCACCATGGATGATGATATCGC CCCTTCTGCCTGACACCTTT  TGTCCTTGGGTGCTGATGTC | TGGTGAAGACGCCAGTGGA  CTGTCCTCCCACTCCAGGTA  AGGCATCTGGTAGAGTCATCAA  CCCATGCTACATTTGCCGAA  GGCAAAACTGCACCTTCACA  AGTGGTGGTCGGAGATTCGT  GCTTGAGGGTTTGCTACAACATG  GCCGGTTCAGGTACTCAGTC  AGCAGTGCCTGTCCAGATTC  AAAGCAGCACCTTTGTGACG  GGCACCAAGTCTGCAAAGTC  CAGTAGGCCAGCGGTAATCT  GGAGGCTAGTGGTCACGTTT  AGACTGTAGGCCACCTCGAT  GAACAGAAAAGGGCTGGGTG  TCTCTGCTCTAACACAGAGGGA  AGGAATCCTTCTGACCCATGC TTCTGTACTGCGGGTGGAAC  TCAGCCCCACACTTCAACAG |
