# Supplementary material for: Fusobacterium nucleatum Facilitates Apoptosis, ROS Generation, and Inflammatory Cytokine Production by Activating AKT/MAPK and NF-κB Signaling Pathways in Human Gingival Fibroblasts
Source: Oxid Med Cell Longev. 2019 Oct 13;2019:1681972. doi: 10.1155/2019/1681972 (PMC6815639; doi:10.1155/2019/1681972)
Supplement: Supplementary 5 — Table 5: top five enriched GO terms in the biological process for the 62 DEGs. [file 1681972.f5.docx]

**Supplementary Table 5:** Top five enriched GO terms in biological process for the 62 DEGs.

| Comparison group | GO annoation | Corrected p-value | Genes |
| --- | --- | --- | --- |
| (T2C vs T2F) vs  (T6C vs T6F) vs  (T12CvsT12F) vs  (T24CvsT24F) vs  (T48CvsT48F) | GO:0002237  response to molecule of bacterial origin | 6.60e-09 | CCL5, NFKBIA, IL6, CCL2, IL1B, PTGS2, ICAM1, CYP27B1, TNFAIP3, CXCL8, GCH1 |
|  | GO:0002376  immune system process | 8.71e-09 | IL1RN, CXCL2, CSF3, CSF2, NFKB2, IRAK2, CD274, IL1B, CCL2, CCL20, CCL5, IL32, IL6, CXCL1, SOD2, CXCL3, ICAM1, RELB, CXCL8, DYRK3, TNFAIP3, NFKBIA |
|  | GO:0009617  response to bacterium | 2.94e-08 | GCH1, CYP27B1, TNFAIP3, CXCL8, ICAM1, PTGS2, CCL2, IL1B, IL6, CCL5, NFKBIA |
|  | GO:0008283  cell proliferation | 4.26e-07 | CSF2, PLAU, CD274, LIF, CSF3, CCL5, IL6, IL1B, CCL2, ADORA2A, CXCL8, CYP27B1, TNFAIP3, CXCL1, SOD2, NFKBIA, CXCL5, INHBA |
|  | GO:0009607  response to biotic stimulus | 2.75e-06 | PTGS2, IL1B, CCL2, IL6, NFKBIA, CCL5, CXCL2, GCH1,CXCL8, TNFAIP3, CYP27B1, ICAM1, |
