# Supplementary figures and images for: Fusobacterium nucleatum Facilitates Apoptosis, ROS Generation, and Inflammatory Cytokine Production by Activating AKT/MAPK and NF-κB Signaling Pathways in Human Gingival Fibroblasts
Source: Oxid Med Cell Longev. 2019 Oct 13;2019:1681972. doi: 10.1155/2019/1681972 (PMC6815639; doi:10.1155/2019/1681972)

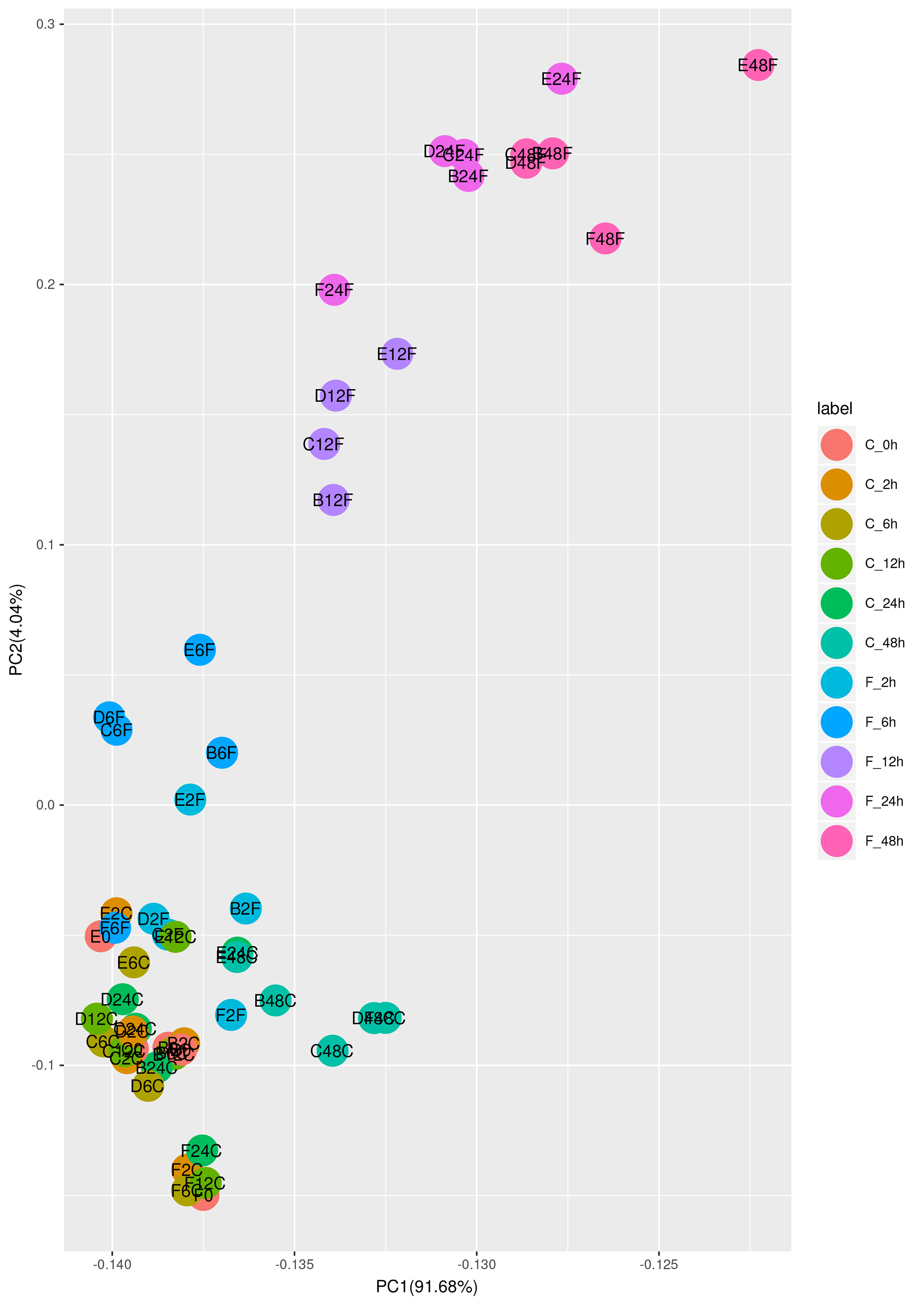

Supplement: Supplementary 6 — Figure 1: PCA of 54 samples of RNA-seq analyses. [file 1681972.f6.tif]

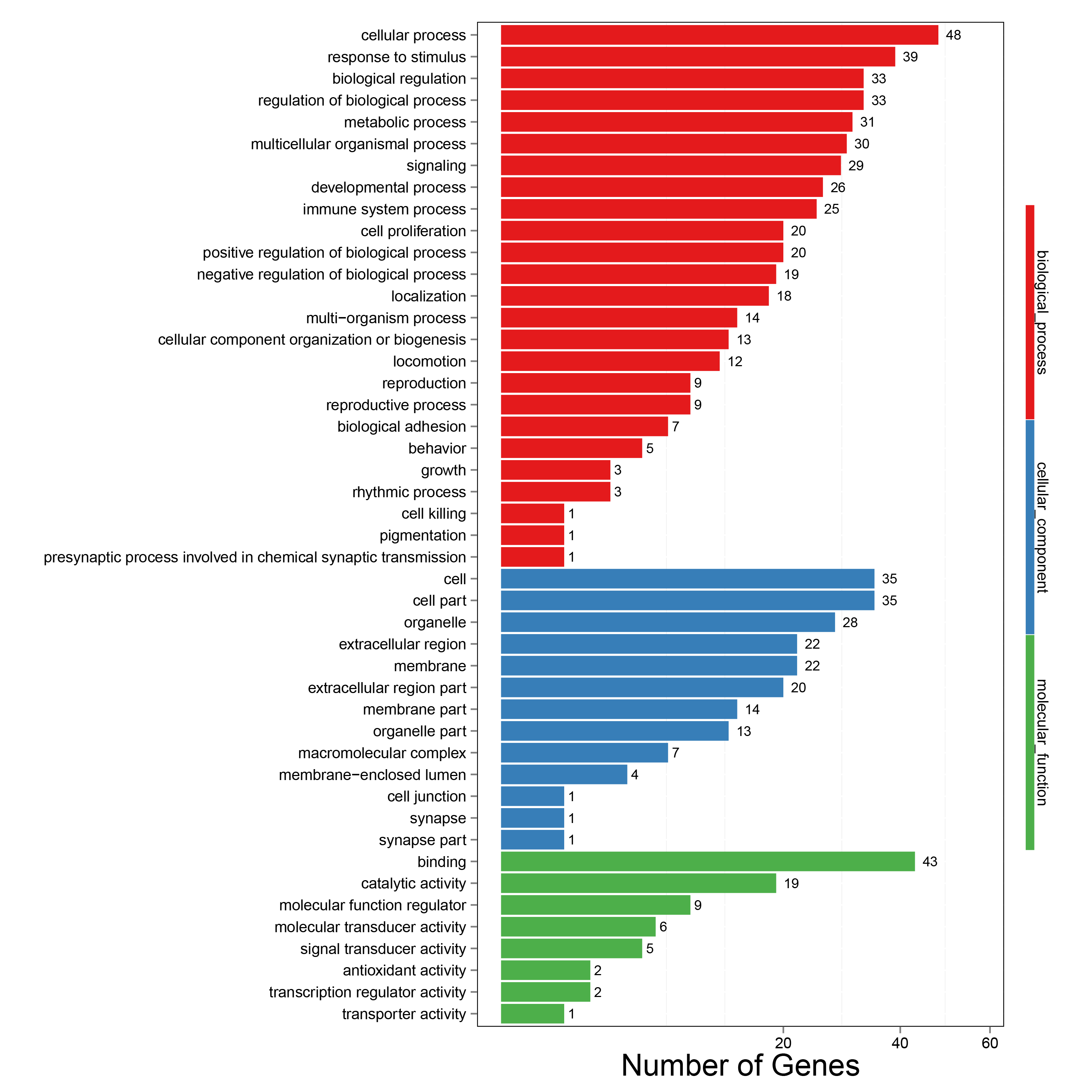

Supplement: Supplementary 7 — Figure 2: GO analysis of the 62 overlapped DEGs after F. nucleatum stimulation at 2 h, 6 h, 12 h, 24 h, and 48 h. [file 1681972.f7.tif]

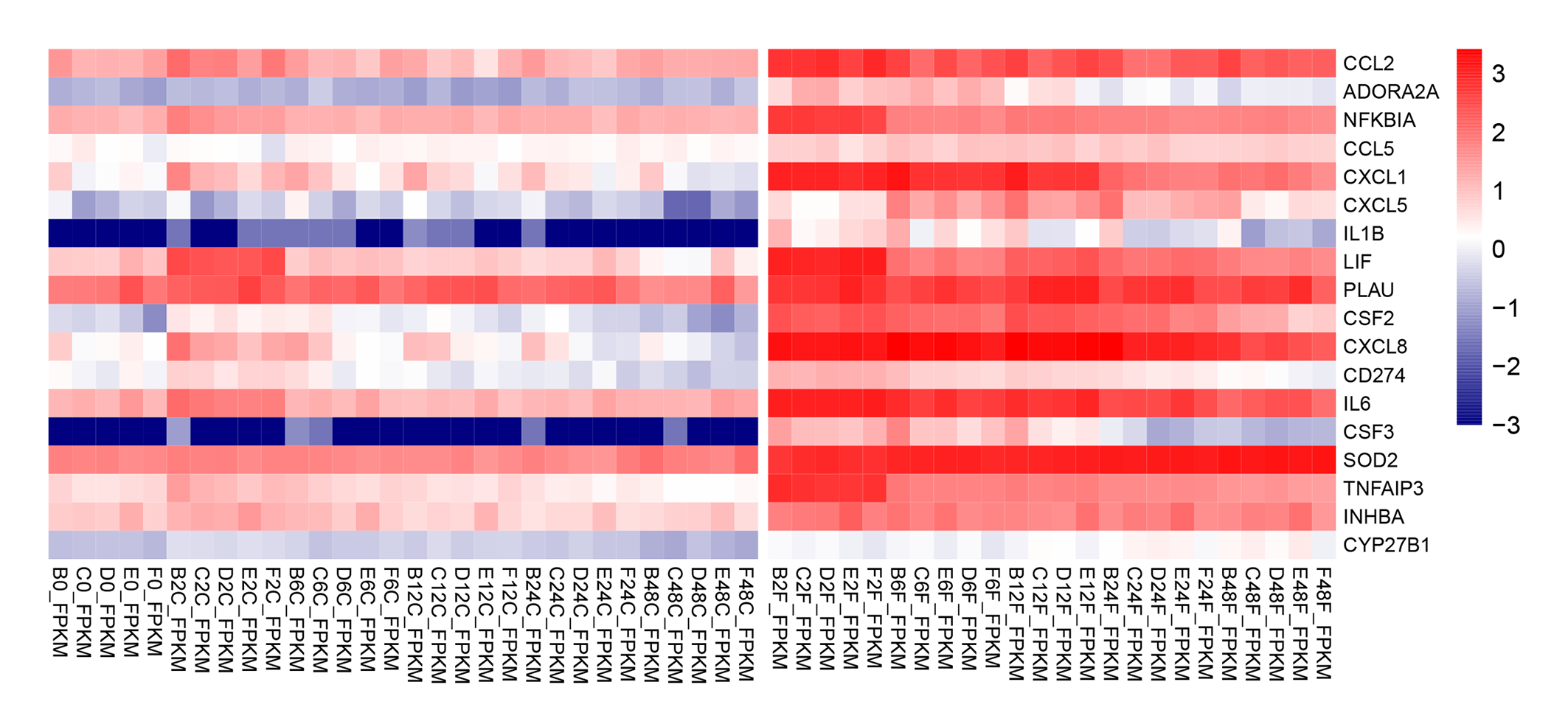

Supplement: Supplementary 8 — Figure 3: heat map summarizing the 18 overlapped DEGs from the GO biological process analysis after the five paired comparisons of cell proliferation. [file 1681972.f8.tif]

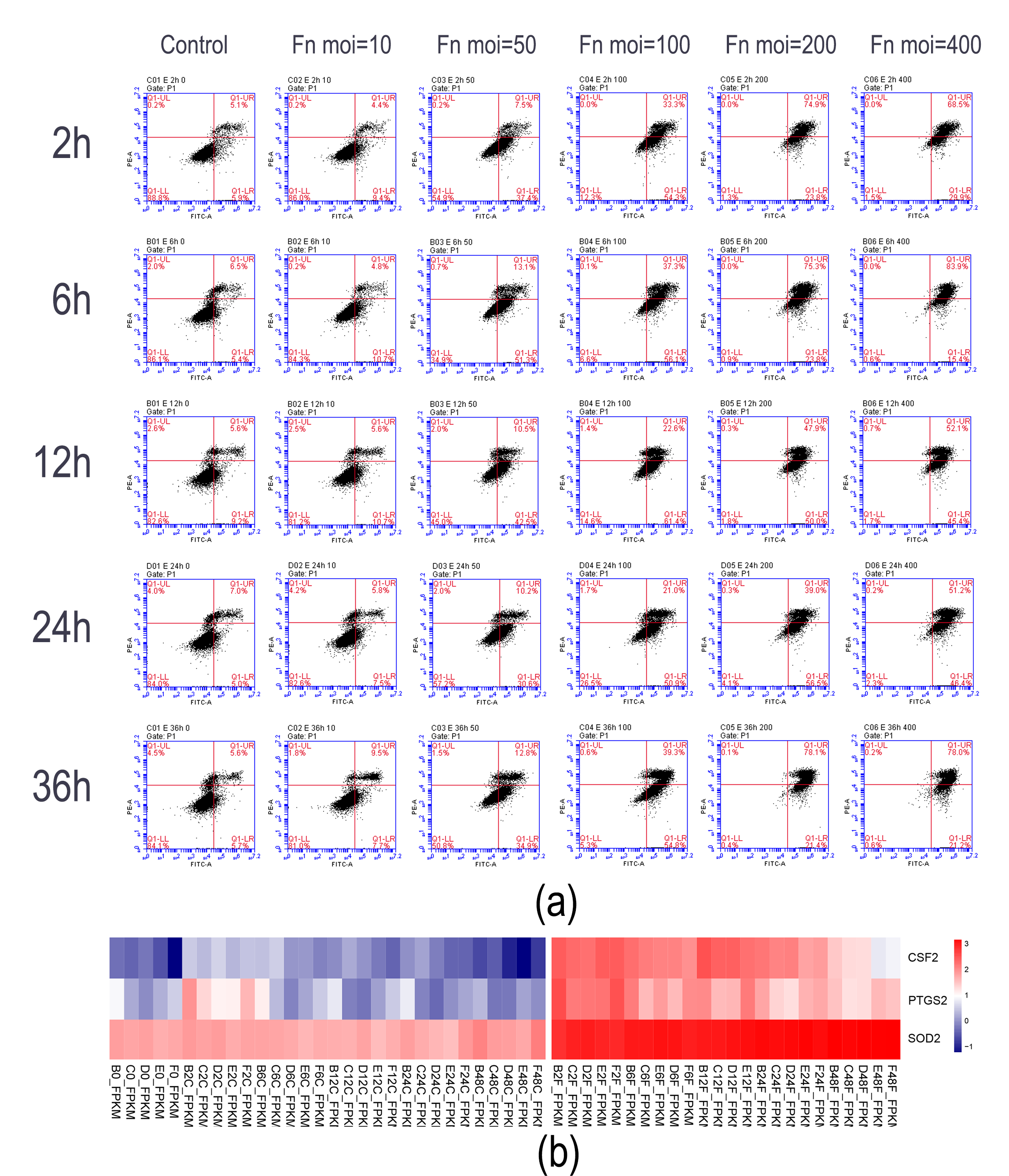

Supplement: Supplementary 9 — Figure 4: (a) Flow cytometry analysis of cell apoptosis. (b) Heat map of the 3 overlapped DEGs from the GO biological process analysis after the five paired comparisons of cell apoptosis. [file 1681972.f9.tif]

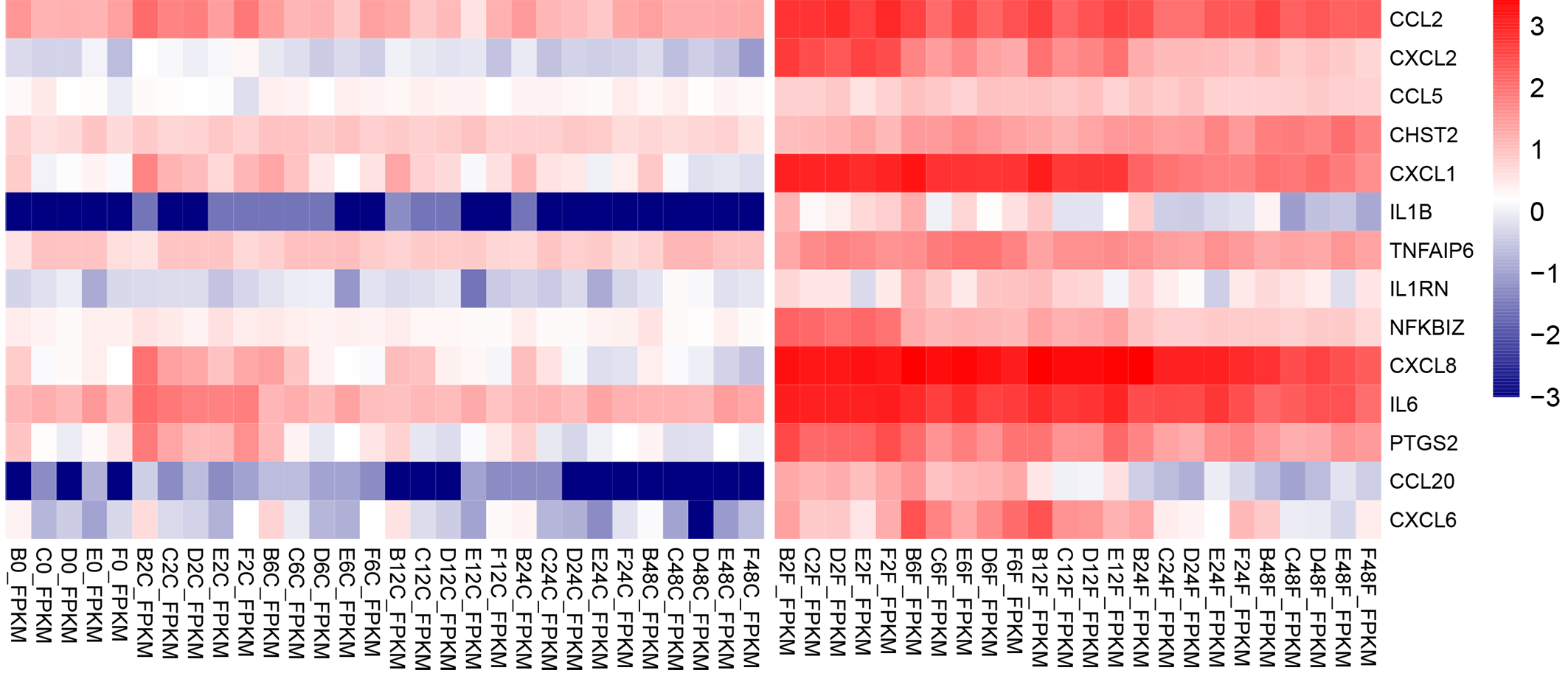

Supplement: Supplementary 10 — Figure 5: heat map of the 14 overlapping DEGs from the GO biological process analysis after the five paired comparisons of cell defense response. [file 1681972.f10.tif]

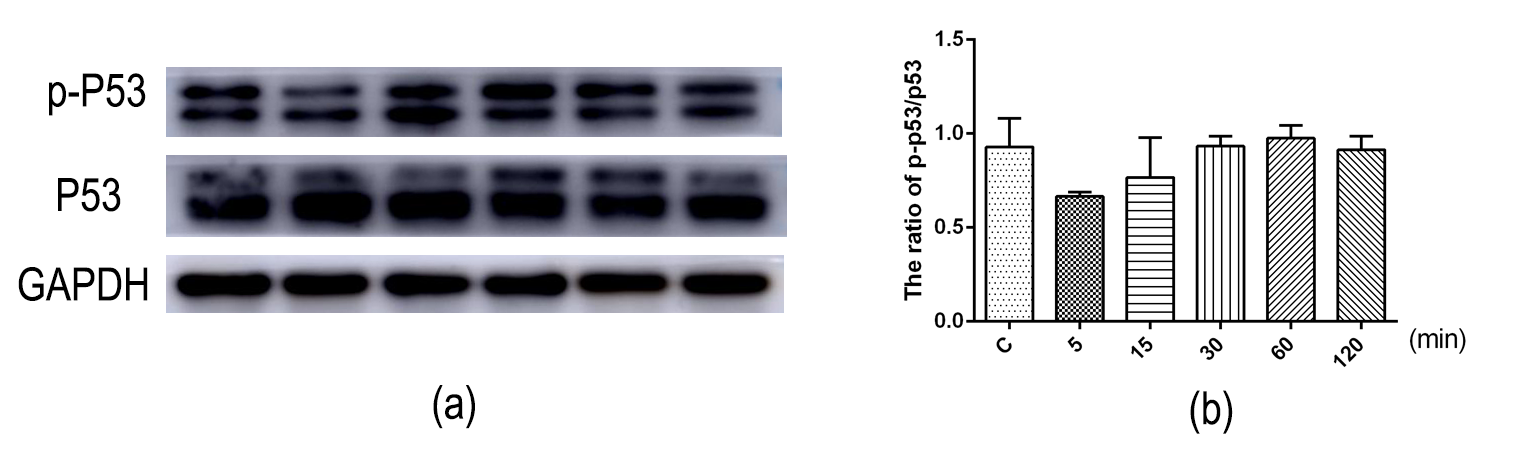

Supplement: Supplementary 11 — Figure 6: (a) The protein levels of p53, phosphorylated p53 (p-p53). (b) The ratio of p-p53 and p53. [file 1681972.f11.tif]

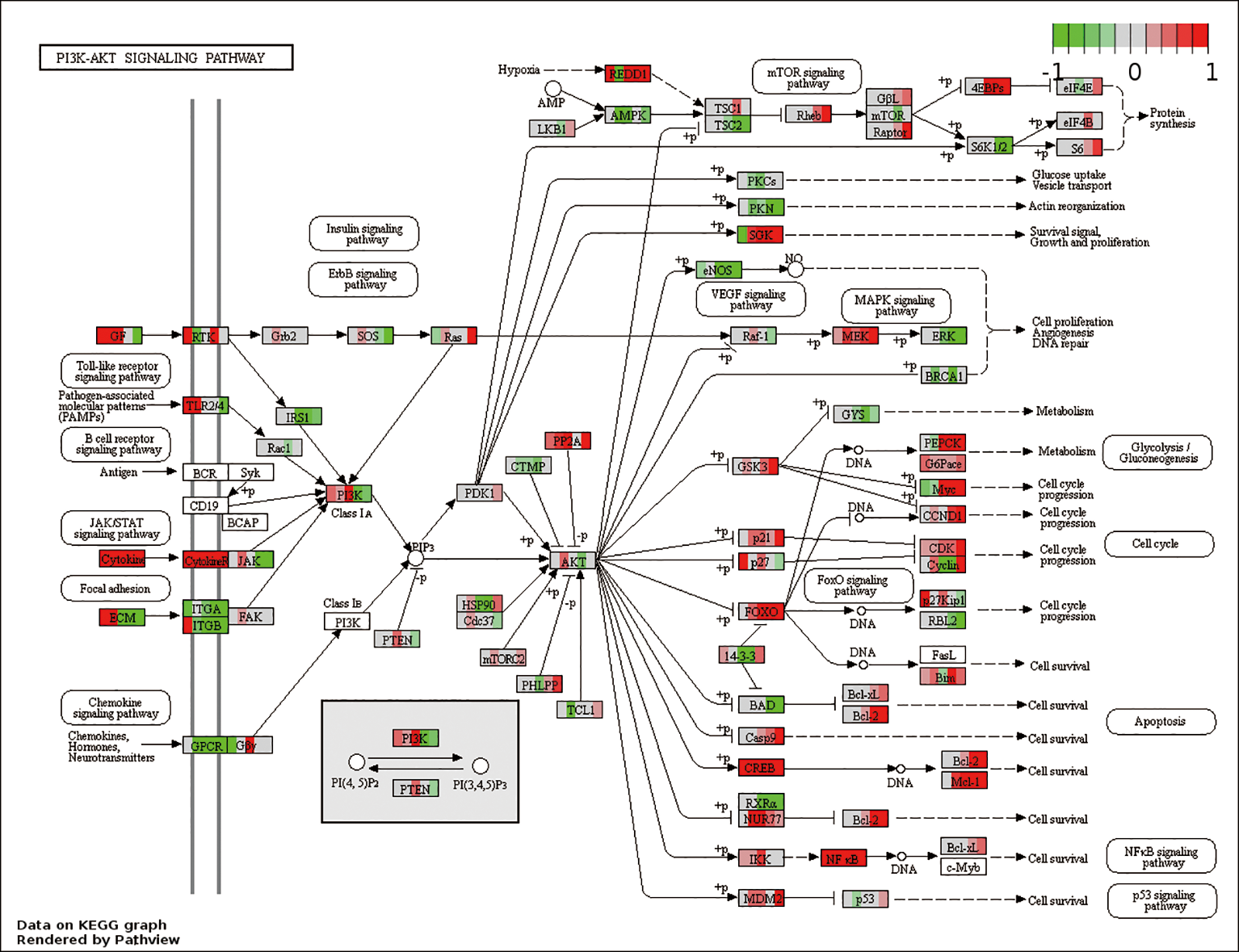

Supplement: Supplementary 12 — Figure 7: the Pathview analysis of the PI3K-AKT signaling pathway. [file 1681972.f12.tif]

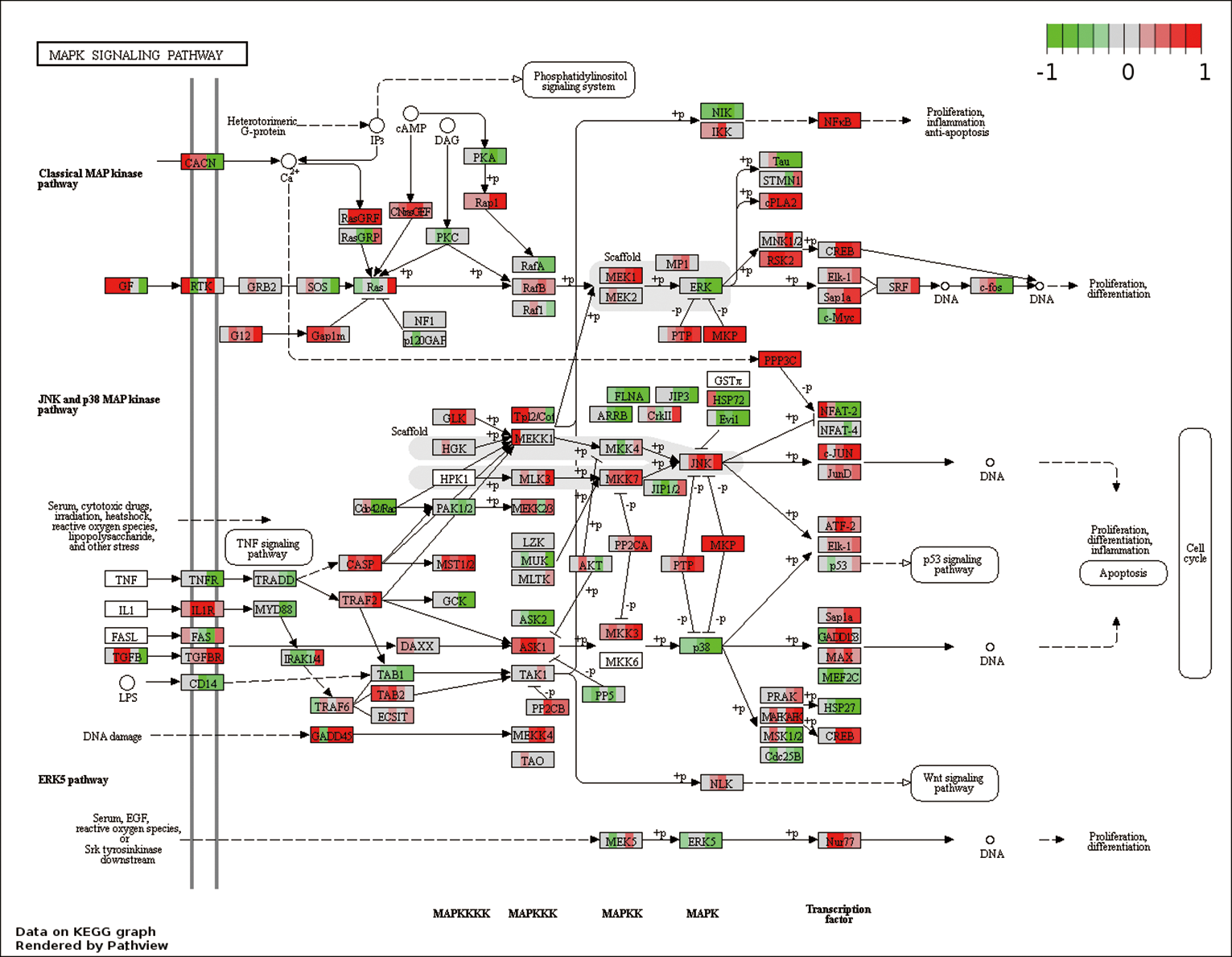

Supplement: Supplementary 13 — Figure 8: the Pathview analysis of MAPK signaling pathway. [file 1681972.f13.tif]

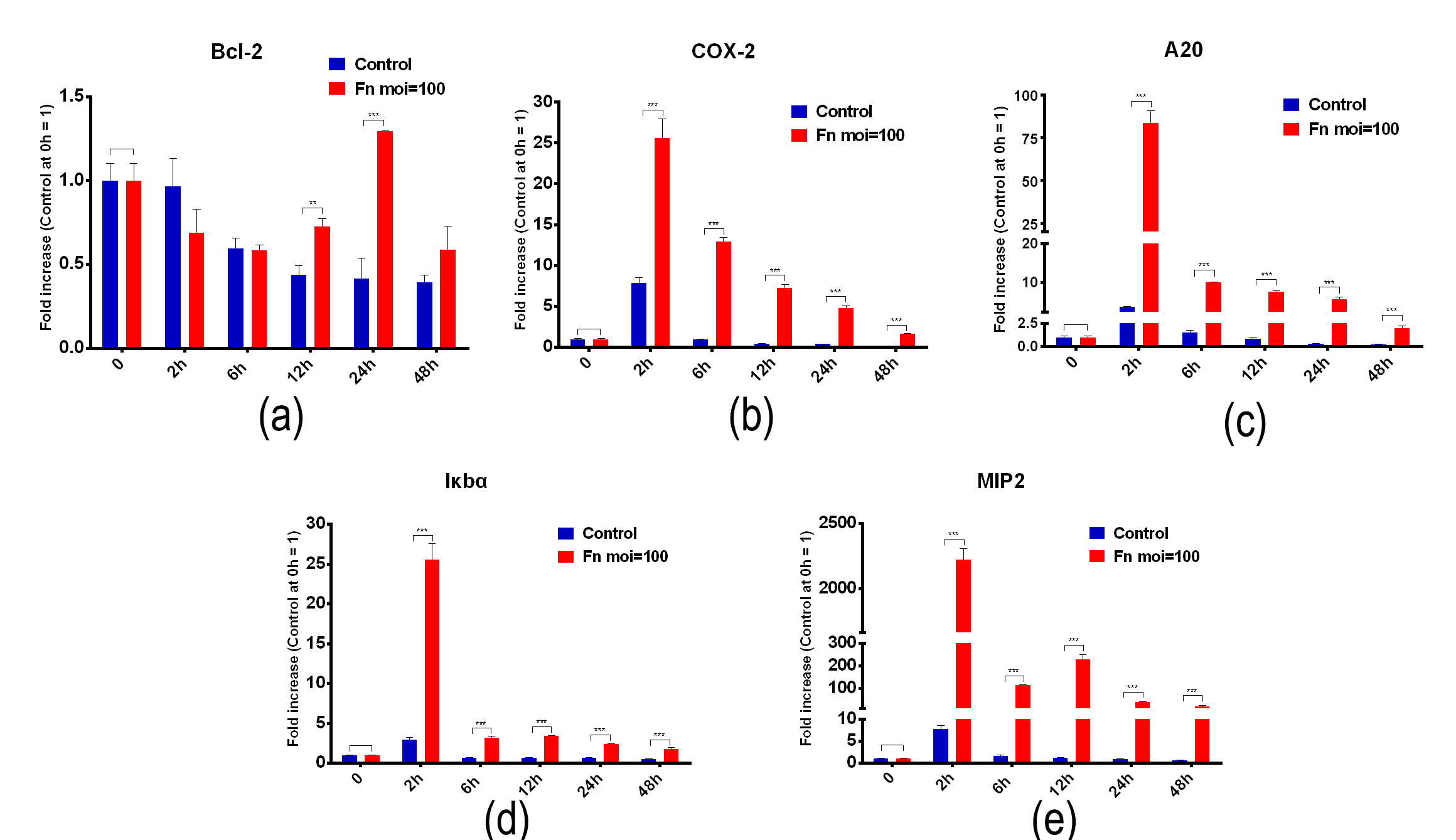

Supplement: Supplementary 14 — Figure 9: the relative gene expression level of Bcl-2 (a), COX2(b), A20 (c), IκBα (d), and MIP2 (e) by qRT-PCR. [file 1681972.f14.tif]

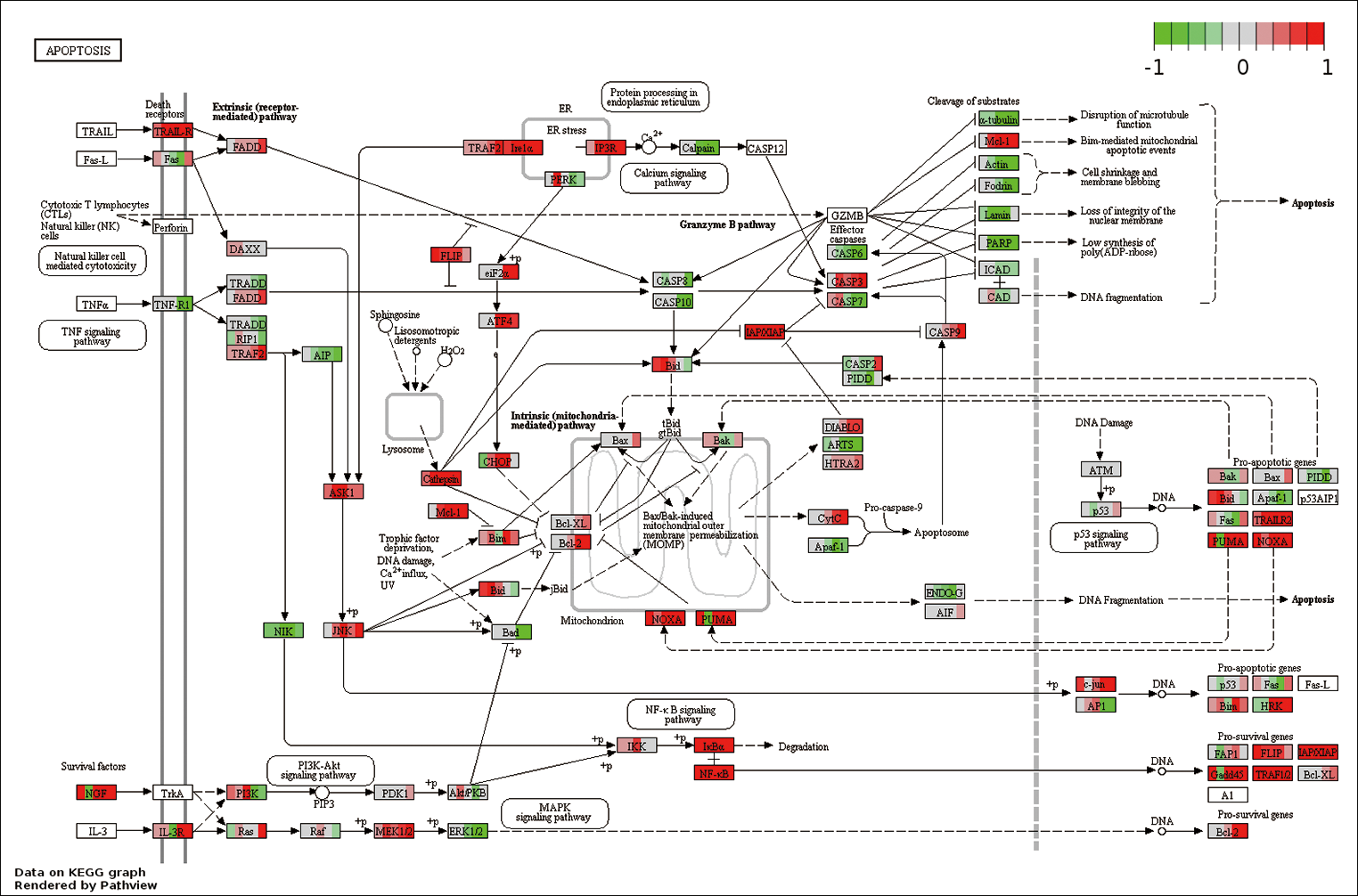

Supplement: Supplementary 15 — Figure 10: the Pathview analysis of the apoptosis signaling pathway. [file 1681972.f15.tif]

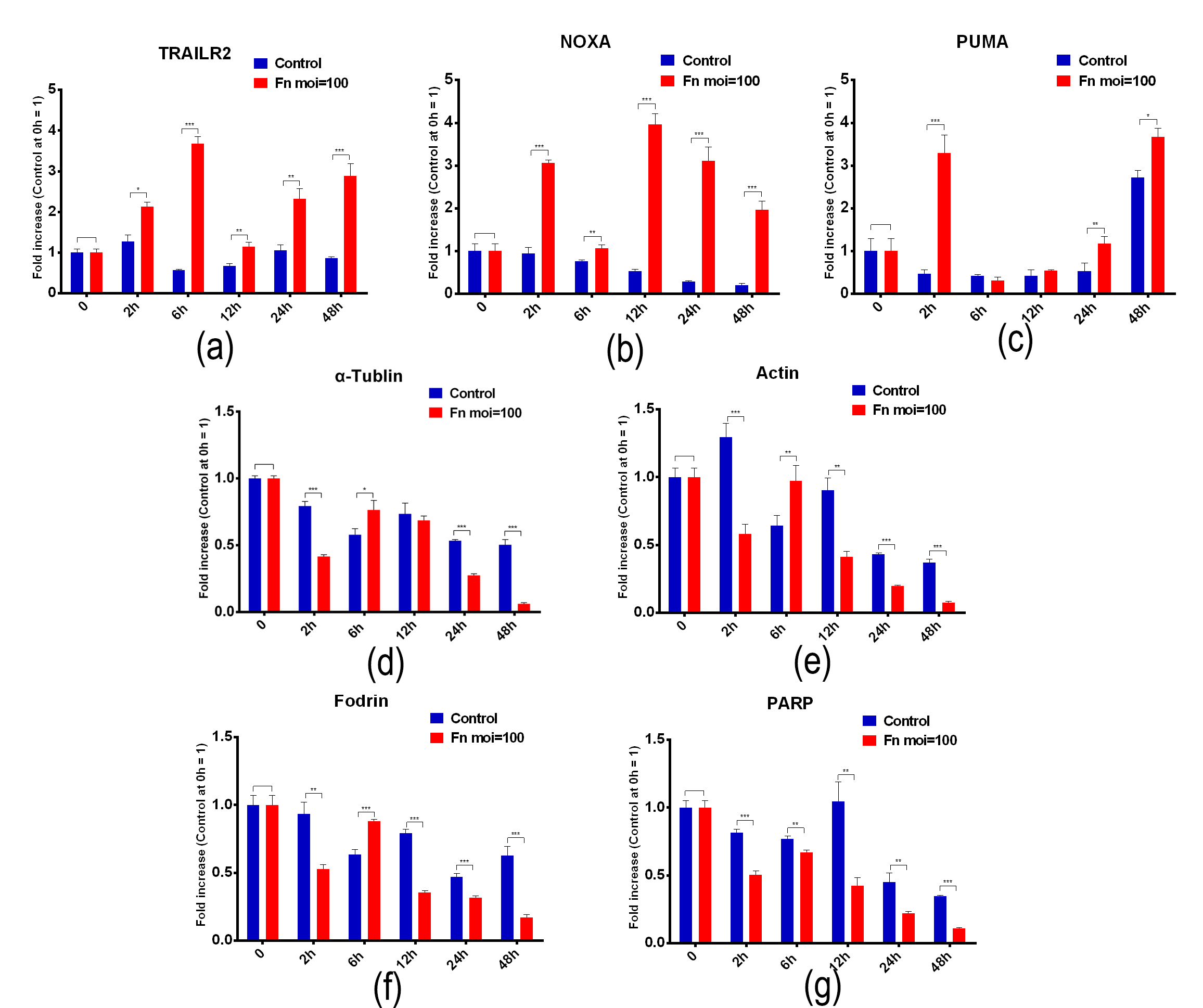

Supplement: Supplementary 16 — Figure 11: the relative gene expression level of TRAILR2 (a), NOXA (b), PUMA (c), tubulin (d), actin (e), fodrin (f), and PARP (g) by RT-PCR. [file 1681972.f16.tif]
